# Tonoplast‐Localized ABCC Transporters Enhance Heterologous Production of the Antidiabetic Montbretin A and Its Analogues in *Nicotiana benthamiana*

**DOI:** 10.1111/pbi.70765

**Published:** 2026-09-28

**Authors:** Charlotte Hijmans, Filipe Cabrita Cruz, Lars H. Kruse, Joerg Bohlmann, Sandra Irmisch

**Affiliations:** ^1^ Plant Sciences and Natural Products, Institute of Biology Leiden University Leiden the Netherlands; ^2^ Michael Smith Laboratories University of British Columbia Vancouver British Columbia Canada; ^3^ Department of Biology Concordia University Montreal Canada

**Keywords:** ABC and MATE transporters, acylated flavonoid glycoside, bioactive metabolite, *Crocosmia* × *crocosmiiflora*, metabolic engineering, montbretia, plant specialized metabolism, synthetic biology, Type 2 diabetes

## Abstract

The plant‐derived specialized metabolite montbretin A (MbA) is under development as an improved treatment for Type 2 diabetes, a worldwide epidemic. MbA is an acylated flavonol glycoside so far only found in small amounts in montbretia (*Crocosmia* × *crocosmiiflora*) corms. The compound's structural complexity and low natural abundance present major challenges for large‐scale production. To enable the development of a scalable MbA production system, we have previously engineered *Nicotiana benthamiana* to produce MbA. In addition to low levels of MbA, *N. benthamiana* also produces the pharmaceutically inactive analogues montbretin B and montbretin C, differing from MbA only in their acyl moieties. Follow‐up research focusing on precursor engineering has improved MbA yield, but further yield optimization is required to establish *N. benthamiana* as a scalable MbA production system. Here, we report evidence that transport likely constrains heterologous montbretin production in *N. benthamiana* and that transporters can be leveraged as bioengineering tools to enhance yield. Specifically, co‐expression of tonoplast localized CcABCC4 transporters with montbretin biosynthetic genes in *N. benthamiana* increased montbretin levels, including those of MbA. Yield increase is likely due to vacuolar sequestration and relies on the presence of an acyl group on the molecule. Eight out of fifteen ABCC4/14 transporters from plant species not known to produce montbretins also enhanced montbretin accumulation in *N. benthamiana*. These observations suggest that ABCC transporters might be versatile tools for bioengineering.

## 1 Introduction

Plants produce an extraordinary diversity of specialized metabolites that constitute a major reservoir of bioactive compounds, many of which have proven or promising new applications in medicine (De 2023). However, the development and use of these compounds as pharmaceuticals is often constrained by supply limitations. Many specialized metabolites are produced only in low quantities, often restricted to a small number of plant species, and exhibit considerable structural complexity (Osbourn and Lanzotti 2009; Pichersky and Lewinsohn 2011; Moghe and Kruse 2018). As a result, large‐scale metabolite production through direct extraction from the original plant source or via chemical synthesis often remains unfeasible.

Metabolic engineering of heterologous systems offers a compelling alternative for producing rare specialized metabolites of interest (Courdavault et al. 2021). Here, complete plant biosynthetic pathways are reconstructed in tractable host organisms, including plants and microorganisms. Following initial proof‐of‐concept for heterologous metabolite production, there is typically a need for yield optimization through refined bioengineering. Traditionally, optimization efforts have primarily focused on enzyme engineering and improving precursor supply (Keasling 2010; Holtz et al. 2024). In addition, more recent research highlights transport processes as critical determinants of metabolic flux, product localization, and biosynthetic yield (Shitan and Yazaki 2013; Shitan 2016; Demurtas et al. 2019; Gharat et al. 2025; Xiao et al. 2025).

In plants, specialized biosynthetic pathways are often distributed across multiple cell types and subcellular compartments, and end products are frequently sequestered away from the site of synthesis to avoid feedback inhibition and toxicity (Döll et al. 2021; Ozber and Facchini 2022; Shen et al. 2024; Vu et al. 2024). Consequently, efficient metabolite accumulation requires transport of intermediates and/or end products in plants. Although some metabolites may efficiently pass across cellular or subcellular membranes through passive diffusion, plants have evolved transporter proteins to mediate and regulate selective metabolite transport as part of compartmentized biosynthetic systems (Shitan 2016; De Brito Francisco and Martinoia 2018). A requirement for selective transport may also apply for effective production of plant metabolites in engineered heterologous systems (Wang et al. 2016; Srinivasan and Smolke 2020; Xiao et al. 2025), which makes metabolite transporters powerful tools for metabolic engineering to regulate and optimize product yield. For example, biosynthesis of the artemisinin precursor dihydroartemisinic acid (DHAA) in *Artemisia annua* occurs in leaf glandular trichomes (Brown 2010). DHAA is then exported to the subcuticular space, where it is converted into artemisinin. Heterologous co‐expression of DHAA biosynthetic genes with the putative DHAA transporters, AaLTP3 and AaPDR2 increased apoplastic accumulation of DHAA and enhanced overall flux through the artemisinin pathway in engineered *Nicotiana benthamiana* (Wang et al. 2016). The biosynthesis of many alkaloids also involves multiple subcellular compartments, cell types, and tissues, requiring transporter‐mediated translocation of intermediates and end products (Kohnen‐Johannsen and Kayser 2019). For the therapeutically valuable alkaloids hyoscyamine and scopolamine, co‐expression of the tonoplast‐localized transporter NtJAT1 from *Nicotiana tabacum* increased yields in engineered yeast, likely by facilitating vacuolar sequestration of pathway intermediates for further biosynthetic processing (Morita et al. 2009; Srinivasan and Smolke 2020).

The discovery of transporters for specific target metabolites, however, remains a significant challenge. This is primarily due to the inherent difficulties associated with studying membrane proteins, the lack of robust substrate prediction tools, transporter promiscuity, and genetic redundancy (Chen et al. 2015; Hwang et al. 2016; Demurtas et al. 2019). In plants, the ATP‐binding cassette (ABC) and the multidrug and toxic compound extrusion (MATE) transporter families have been identified as key players in the transport and compartmentation of specialized metabolites (Omote et al. 2006; Kang et al. 2011; Kretzschmar et al. 2011; Upadhyay et al. 2019). In *Coptis japonica*, for instance, CjABCB1 and CjABCB2 facilitate the transport of berberine across the plasma membrane into the cytosol, thereby enabling its translocation from the root to the rhizome (Shitan et al. 2003, 2013). From the cytosol, berberine is subsequently sequestered into the vacuole for storage, likely via CjMATE1 (Takanashi et al. 2017). Both ABCs and MATEs are large and structurally diverse protein families with often broad substrate specificity (Hwang et al. 2016; Lefèvre and Boutry 2018; Upadhyay et al. 2019).

The specialized plant metabolite montbretin A (MbA), a complex acylated flavonol glycoside, is currently being developed as a novel treatment option for Type 2 diabetes, which has been named a “defining disease of the 21st century” due to its worldwide epidemic scale (Tarling et al. 2008; Yuen et al. 2016; The Lancet 2023). MbA represents a promising alternative to current blood glucose‐lowering treatments, which often cause undesirable side effects. This is due to MbA's highly specific and potent (K_i_ of 8 nM) inhibition of human pancreatic α‐amylase (HPA), a key enzyme in starch digestion (Tarling et al. 2008; Williams et al. 2015). Toxicity and efficacy have previously been evaluated in rodent models (Yuen et al. 2016), and Phase 1 clinical trials are currently underway (Petrella 2024).

To date, the only known source of MbA is the ornamental plant montbretia ( *Crocosmia* × *crocosmiiflora* ) (Tarling et al. 2008). Montbretia plants contain MbA in the underground bulb‐like storage and reproductive organs, called corms. Scalable MbA production is not feasible using extraction from plants or chemical/chemo‐enzymatic synthesis due to low MbA levels in corms and the compound's chemical complexity, respectively. As an alternative, we are exploring engineered heterologous hosts for MbA production (Irmisch et al. 2018, 2020; Irmisch, Jancsik, et al. 2019; Irmisch, Ruebsam, et al. 2019; Sunstrum et al. 2021; Kruse et al. 2024). In previous work, we have elucidated the six‐step MbA assembly pathway in montbretia and reconstructed MbA biosynthesis in *N. benthamiana*, providing proof‐of‐concept for MbA production in heterologous systems. In addition to MbA, engineered *N. benthamiana* also produces the two analogues, montbretin B (MbB) and montbretin C (MbC), which harbour a coumaroyl and feruloyl moiety, respectively, instead of a caffeoyl moiety. Importantly, MbB and MbC are not effective as HPA inhibitors, with K_i_‐values of 3600 and 6100 nM, respectively (Tarling et al. 2008; Williams et al. 2015). However, in engineered *N. benthamiana*, MbB was about 50‐fold more abundant than MbA (Irmisch et al. 2020; Kruse et al. 2024), with MbB formation directly competing for metabolite flux towards MbA. Follow‐up strategies to increase MbA yields have included bioengineering of flavonoid and caffeoyl‐CoA precursor supply (Irmisch, Jancsik, et al. 2019; Kruse et al. 2024). Building upon this foundation that proved the feasibility of MbA metabolic engineering, the scalable production of MbA in *N. benthamiana* requires further optimization.

Following their biosynthesis, flavonoid glycosides are typically sequestered into the plant vacuole, and members of the ABC and MATE transporter families have been implicated in their transport (Gomez et al. 2009; Pourcel et al. 2010; Behrens et al. 2019; Kitashova et al. 2024). We therefore hypothesized that the accumulation of the acylated flavonoid glycoside MbA and other montbretins involves tonoplast‐localized transporters of the ABC or MATE family, and that these transporters could be leveraged as bioengineering tools to improve MbA production in heterologous systems. In this work, we identified ABC and MATE transporters from montbretia and describe their characterization and subcellular localization when expressed in MbA‐producing *N. benthamiana*. Expression of tonoplast‐localized CcABCC4 transporters increased the accumulation of MbA, as well as MbB and MbC (collectively referred to as montbretins), which is likely due to vacuolar sequestration. Additionally, partial pathway reconstruction experiments showed that yield increase is only observed with intermediates and products of the montbretin pathway that include the acyl moiety, suggesting that substrate recognition and transport may be dependent on the acylation of the molecule. Furthermore, ABCC transporter homologues from several plant species not known to produce montbretins also enhanced montbretin accumulation, indicating a degree of substrate promiscuity within the ABCC transporter family.

## 2 Results

### 2.1 Candidate ABC and MATE Transporters Are Co‐Expressed With Montbretin Biosynthetic Genes During Early Corm Development

To identify montbretia transporters potentially involved in the transport of MbA, candidate CcABC and CcMATE transporter sequences were identified in the montbretia corm transcriptomes using a BLASTP search (Irmisch et al. 2018, 2020). In total, we found 75 putative CcABC transporters, which clustered into the subfamilies A to G (Figure S1). Subfamilies B, C and G held the most representatives in the montbretia transcriptome, with 20, 14, and 30 members, respectively. Given that ABCC transporters are generally localized to the tonoplast (Kang et al. 2011; De Brito Francisco and Martinoia 2018; Demurtas et al. 2019), the ABCC subfamily was prioritized for candidate selection (Figure 1A). Additional candidates were chosen from subfamilies B and G (Figure S1). All candidates were selected based on higher expression in early compared to late corm development, and strong correlation (*r* ≥ 0.8) to montbretin biosynthetic pathway genes (*CcUGT1‐5* and *CcAT1*) in gene expression during corm development (Figure 1, Table S1). Altogether, two CcABCCs (*CcABCC4A* and *CcABCC4B*), two CcABCBs (*CcABCB1B* and *CcABCB19*) and two CcABCGs (*CcABCG28B* and *CcABCG31B*) were chosen for characterization.

**FIGURE 1:**
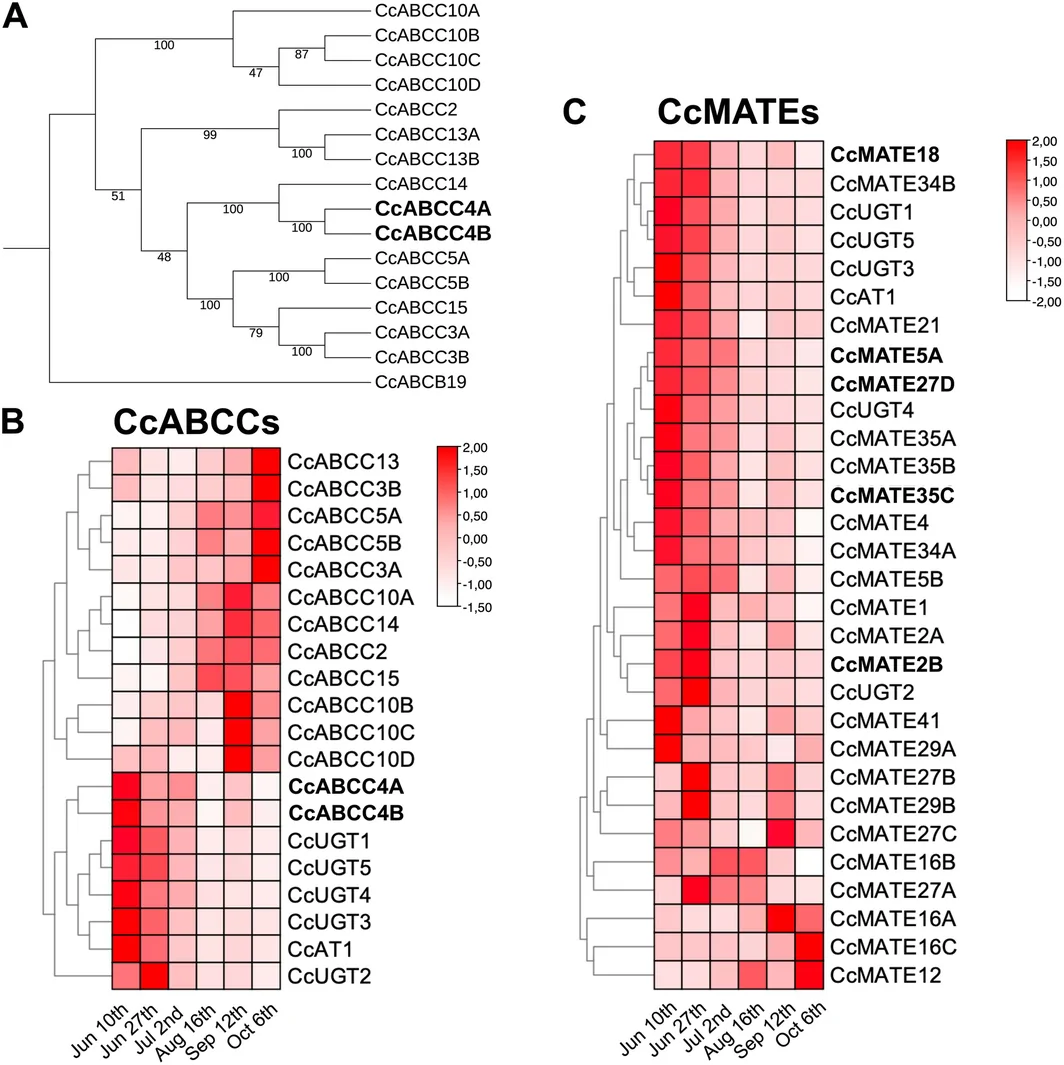
ABCC and MATE transporters expressed in montbretia corms. (A) Phylogenetic analysis of the ABCC transporter subfamily expressed in montbretia corms. The predicted amino acid sequences were identified using a corm transcriptome and aligned using the MUSCLE algorithm in Geneious Prime. The maximum‐likelihood phylogeny was constructed using IQTree and visualized in iTOL. CcABCB19 was used to root the tree. Bootstrap values (ultrafast) are shown at each node. (B, C) Heatmaps showing the co‐expression (in counts per million, cpm) of CcABCCs (B) and CcMATEs (C) with montbretin biosynthetic genes throughout corm development. Transporters characterized in this work are in bold. *Cc, Crocosmia* × *crocosmiiflora* .

Thirty putative CcMATE transporter sequences, including members of all four subfamilies (I‐IV), were identified in the montbretia corm transcriptomes (Figure S2). Subfamily II harbours more than half of the identified MATE candidates (17 CcMATEs), whereas clades I, III, and IV only contain seven, four, and two representatives, respectively. Each MATE subfamily can be subdivided into smaller subgroups, of which the number varies per subfamily (Nimmy et al. 2022). Predicted CcMATE aa sequences clustered in all but the two subgroups IVA and IVB. Five CcMATEs, which had ≥ 5‐fold higher expression in early compared to late corm development (Table S1) and showed high correlation (*r* ≥ 0.9) in expression patterns to MbA biosynthetic genes (*CcUGT1‐5, CcAT1*) over corm development, were chosen for further characterization. These candidates were designated CcMATE2B, CcMATE5A, CcMATE18, CcMATE27D, and CcMATE35C and cluster into subfamilies IIA, IB, IA, IIE, and IIA, respectively.

Full‐length open reading frames (ORFs) for all six CcABC sequences were amplified from montbretia corm cDNA. Translated aa sequences of CcABCB1B and CcABCB19 shared about 55% aa pairwise identity. The two proteins (and CDS) of CcABCC4A1 and CcABCC4A2 shared approximately 98% aa (and nt) sequence identity and likely represent allelic variants or products of a recent gene duplication event. The two CcABCC4As and CcABCC4B share approximately 82% aa sequence identity. CcABCG28B and CcABCG31B share about 36% aa pairwise identity. In addition, full‐length ORFs for all five CcMATE candidates, CcMATE2B, CcMATE5A, CcMATE18, CcMATE27D, and CcMATE35C were amplified and their translated aa sequences share between 26% and 40% pairwise aa identity. Expression levels in young developing corms—the organs of active MbA biosynthesis—were highest for CcABCC4A1/2 (215 counts per million, cpm) compared to CcABCC4B (58 cpm), MATE2B (11 cpm), MATE5A (5 cpm), MATE18 (10 cpm), MATE27D (10 cpm) and MATE35C (132 cpm) (Table S1).

### 2.2 Expression of Transporters Increases Heterologous Montbretin Production Levels in *N. benthamiana*

In previous work, we transiently engineered *N. benthamiana* for the heterologous production of MbA (Irmisch et al. 2018, 2020; Irmisch, Jancsik, et al. 2019; Irmisch, Ruebsam, et al. 2019). Transformed plants also produced the pharmaceutically inactive analogues MbB and MbC. Additional engineering of precursor pathways resulted in increased MbA levels, but MbB remained the dominant product (Kruse et al. 2024). We further improved the transformation and expression system by combining the 11 genes used for MbA bioengineering into two multi‐gene constructs (MGCs) (Kruse et al. 2024). In the present work, we used this improved *N. benthamiana* system to study the effect of candidate transporter expression on MbA accumulation and also monitored effects on MbB and MbC levels. *N. benthamiana* plants were co‐infiltrated with the two MGCs carrying the montbretin biosynthetic genes and a pCAM plasmid encoding a montbretia transporter candidate. Control plants were infiltrated with the MGCs only. Leaves were harvested 5 days post‐infiltration, followed by metabolite extraction and liquid chromatography–mass spectrometry (LC–MS) analysis. MbA, MbB, and MbC (Figure 2A) were quantified individually, and the sum of these three metabolites represents total montbretin production.

**FIGURE 2:**
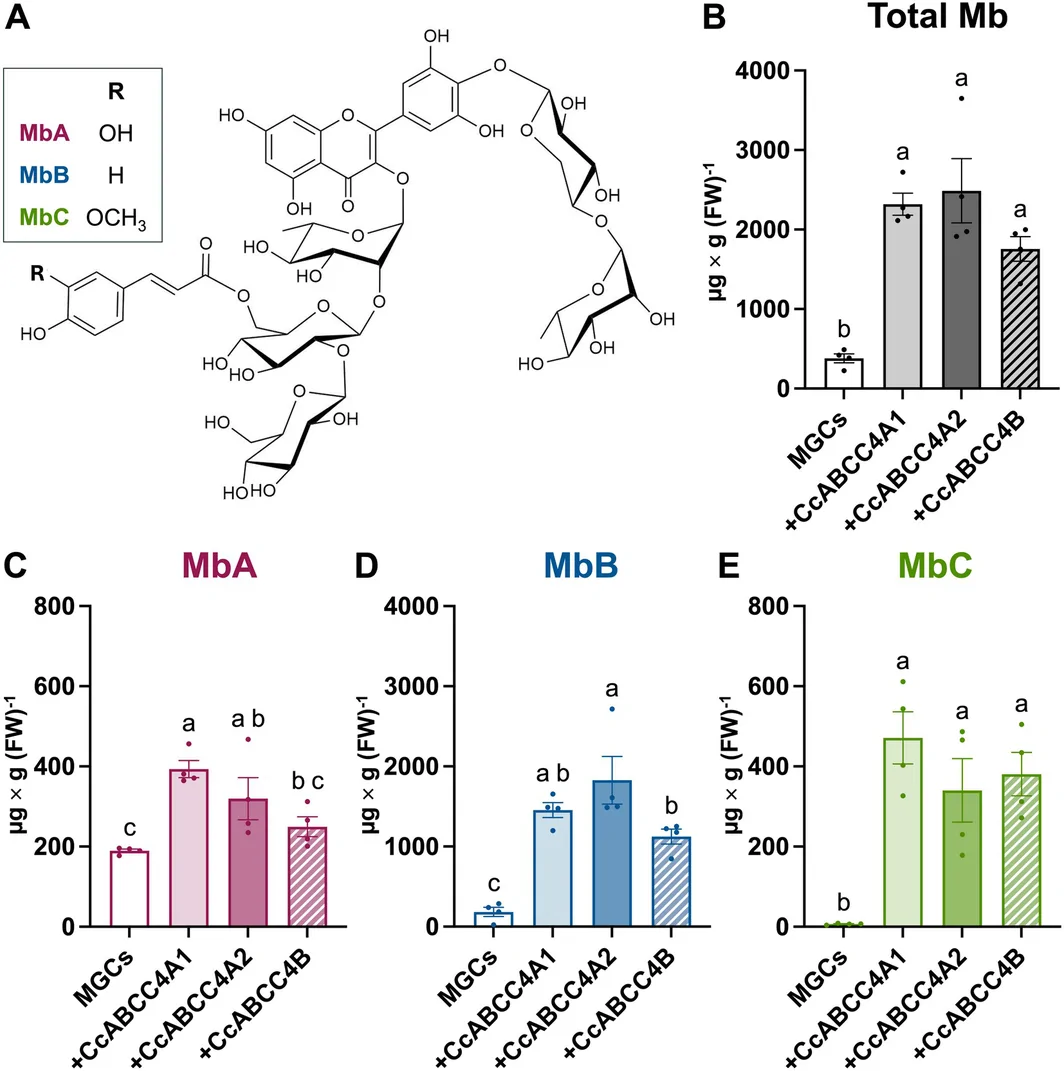
Expression of CcABCC4 transporters increases montbretin accumulation in *N. benthamiana*. (A) Chemical structure of different montbretins. (B–E) Montbretins produced by engineered *N. benthamiana*. Leaves of *N. benthamiana* transiently expressing montbretin biosynthesis genes with and without co‐expressing a transporter gene were harvested 5 days after infiltration. Extracts were analysed using LC–MS and total montbretin (Mb; MbA + MbB + MbC; B), MbA (C), MbB (D) and MbC (E) levels were quantified using an external MbA standard curve. Mean and standard error of four biological replicates (*n* = 4) are plotted, and individual data points are shown as dots. Different letters indicate statistically significant differences between groups (one‐way ANOVA followed by Tukey's HSD post hoc test, *p* < 0.05; Table S7, Data S5). Cc, *Crocosmia* × *crocosmiiflora* ; FW, fresh weight; Mb, montbretins; MGC, multi‐gene construct.

Co‐expression of CcABCC4A1, CcABCC4A2, or CcABCC4B with MGCs resulted in a significant increase in total montbretin levels of about 6‐, 7‐, and 5‐fold, respectively, as compared to the control not expressing a transporter gene (Figure 2B). Notably, expression of CcABCC4A1 or CcABCC4A2 led to a ~2‐fold increase in MbA (Figure 2C), whereas CcABCC4B only had a minor effect on MbA levels. The increase in the total montbretin content was mainly due to higher MbB (~4 to 11‐fold; Figure 2D) and MbC (~31‐ to 53‐fold; Figure 2E) levels. We also evaluated the effect of transporter expression on the accumulation of intermediates in the montbretin biosynthetic pathway. No montbretin intermediates were detected in the control samples or when CcABCC4B was expressed. When CcABCC4A1 or CcABCC4A2 was expressed, we detected low levels of MbA‐XR^2^, MbB‐XR^2^ and MbC‐XR^2^ (Figure S3). Co‐expression of either of the two CcABCB or the two CcABCG candidates with the MGCs did not alter montbretin or montbretin intermediate levels compared to the control (Figure S4).

Out of the five CcMATE candidates, only co‐expression of CcMATE18 with MGCs resulted in a ~2‐fold increase in total montbretin content compared to the control (Figure 3). Here, only MbB but not MbA levels increased significantly. Expression of CcMATE2B decreased montbretin levels to trace amounts, while expression of the other three MATE transporters did not change montbretin levels. No montbretin intermediates were detected in any of the samples expressing CcMATE transporters.

**FIGURE 3:**
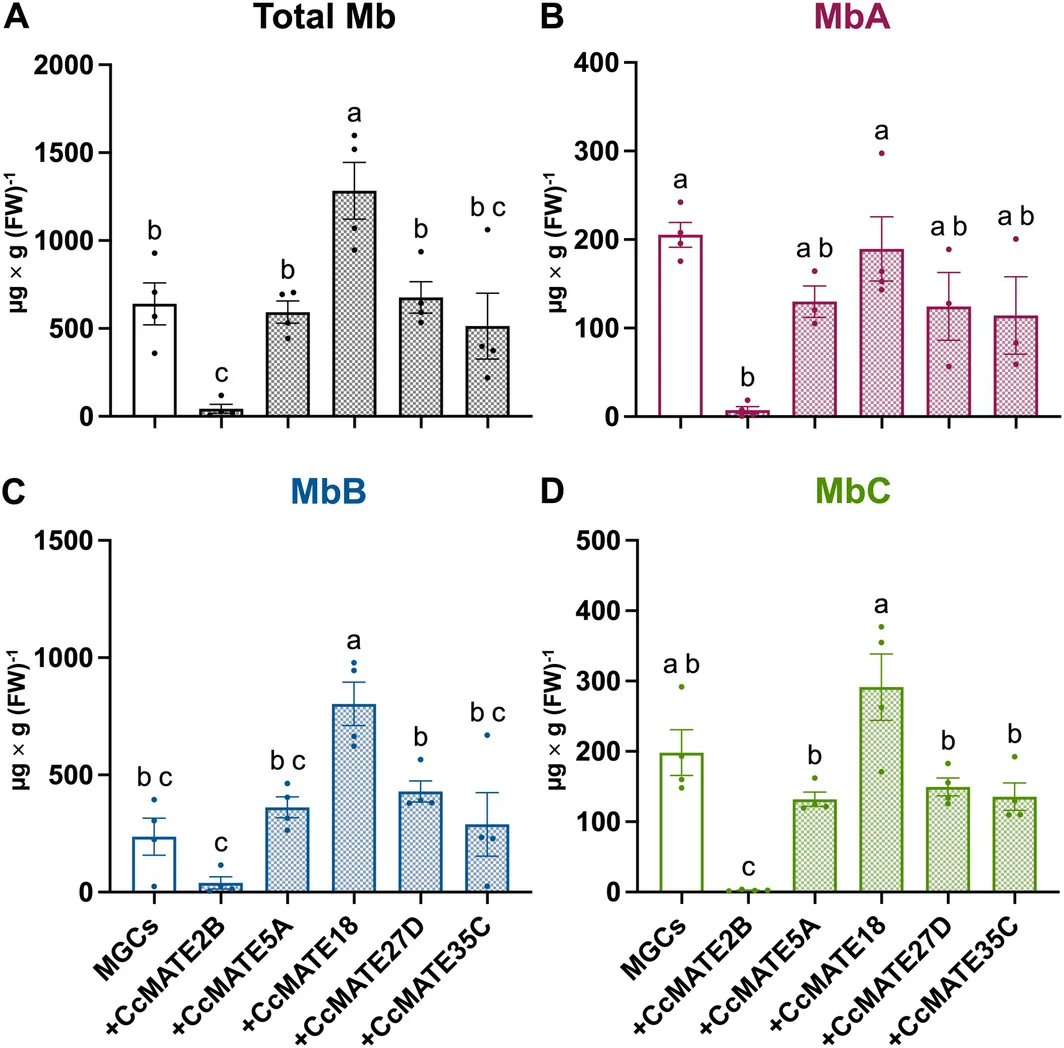
Co‐expression of CcMATE18 with montbretin biosynthesis genes increases montbretin accumulation in *N. benthamiana*. Leaves of *N. benthamiana* transiently expressing the montbretin biosynthesis genes with and without co‐expressing transporter genes were harvested 5 days after infiltration. Extracts were analysed using LC–MS and total montbretin (Mb; MbA + MbB + MbC; A), MbA (B), MbB (C) and MbC (D) levels were quantified using an external MbA standard curve. Mean and standard error of four biological replicates (*n* = 4) are plotted, and individual data points are shown as dots. Different letters indicate statistically significant differences between groups (one‐way ANOVA followed by Tukey's HSD post hoc test, *p* < 0.05; Table S7, Data S5). Cc, *Crocosmia* × *crocosmiiflora* ; FW, fresh weight; Mb, montbretins; MGC, multi‐gene construct.

Overall, plants expressing CcABCC4A1 or CcABCC4A2 accumulated the highest level of total montbretins with ~2.5 mg × g (FW)^−1^, while control plants not expressing a transporter produced ~0.38 mg × g (FW)^−1^ total montbretins (Table S2). MbA levels were increased from 0.19 mg × g (FW)^−1^ in controls to 0.32 mg × g (FW)^−1^ in CcABCC4A2‐expressing plants.

### 2.3 CcABCC4 Transporters Localize to the Tonoplast in *N. benthamiana*

Based on the sequence features of CcUGT and CcAT enzymes involved in montbretin biosynthesis, the MbA assembly pathway is thought to occur in the cytosol (Tables S3 and S4) (Irmisch et al. 2018, 2020; Irmisch, Ruebsam, et al. 2019). To support a proposed role of the identified transporters in vacuolar sequestration of MbA, we studied the subcellular localization of the three CcABCC4s and CcMATE18. From their translated amino acid sequences, DeepLoc 2.1 predicted a localization of the transporters to the plasma membrane or tonoplast and no N‐terminal targeting signal was predicted with TargetP‐2.0 (Tables S3 and S4) (Almagro Armenteros et al. 2019; Nielsen 2025). The transporters were fused to GFP to facilitate visualization in *N. benthamiana* leaves using confocal imaging. γTIP::mRuby2 was used as a tonoplast marker, FM4‐64 was used to stain the plasma membrane, and soluble GFP was used to indicate the cytoplasm (Figure S5). Expression of GFP‐tagged CcABCC4 transporters in *N. benthamiana* leaves that were co‐infiltrated with γTIP::mRuby2 resulted in overlapping GFP and mRuby2 signals (Figure 4A, Figures S6A and S7A). In contrast, expression of GFP‐tagged CcABCC4 transporters and staining with FM4‐64 did not result in overlapping fluorescent signals (Figure 4B, Figures S6B and S7B). These results indicate that the candidate CcABCC4 transporters localize to the tonoplast but not to the plasma membrane in *N. benthamiana* leaves. For CcMATE18, we observed overlap of the fluorescence signal with γTIP::mRuby2 as well as with the FM4‐64, indicating that CcMATE18 localizes to both the plasma membrane and the tonoplast (Figure S8A,B). Co‐localization analysis of pixels of both fluorescent signals further supports tonoplast (*r* = 0.58–0.63) localization for the three CcABCC4s, and CcMATE18 localization to the tonoplast (*r* = 0.62) and plasma membrane (*r* = 0.58; Table S5).

**FIGURE 4:**
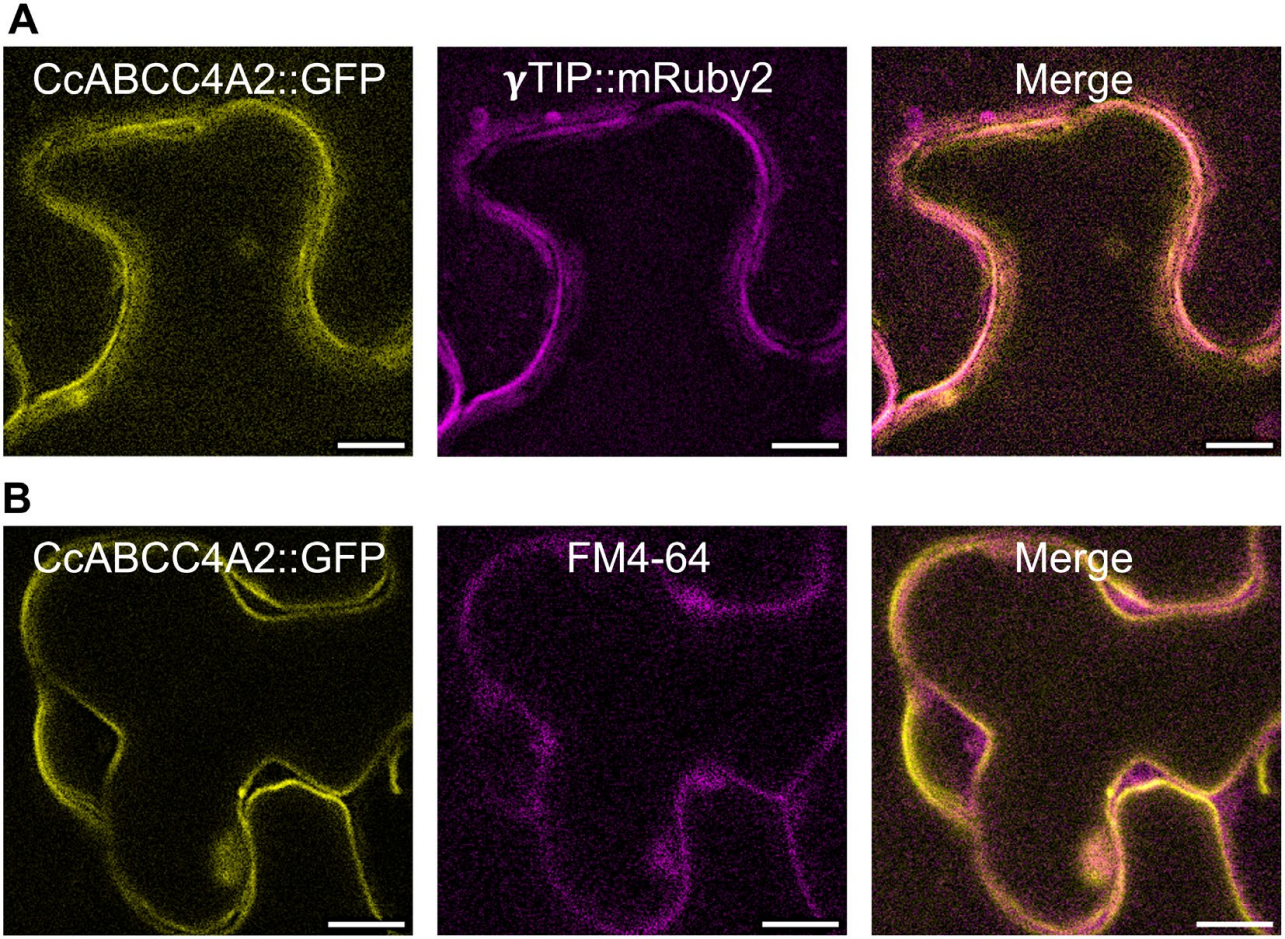
CcABCC4A2 localizes to the tonoplast in *N. benthamiana* leaves. GFP was fused to the C‐terminus of CcABCC4A2 and transiently expressed in *N. benthamiana*. Leaf tissue was subjected to confocal microscopy 5 days after infiltration to visualize the subcellular transporter localization. The tonoplast marker *AtγTIP*::*mRuby2* was co‐infiltrated with *CcABCC4A2*::*GFP* (A) and FM4‐64 was used to stain the plasma membrane of plants expressing *CcABCC4A2*::*GFP* (B). Representative images of at least three biological replicates are shown. Scalebars measure 50 μm.

C‐terminally GFP‐tagged CcABCC4s and the N‐terminally GFP‐tagged CcMATE18 remained functional as co‐expression with montbretin biosynthetic genes led to increased montbretin levels compared to the control, although to a lesser extent compared to the untagged transporters (Figures S9 and S10).

### 2.4 Acylation Influences Metabolite Accumulation in the Presence of CcABCC4 Transporters

In addition to the three montbretins, MbA, MbB, and MbC, we tested if CcABCC4 transporters also affect accumulation of intermediates in the montbretin biosynthetic pathway. For this purpose, we reconstituted truncated versions of the montbretin biosynthetic pathway up to the respective intermediate and co‐expressed one of the CcABCC transporters in *N. benthamiana* (Table S6). Plants expressing the truncated pathways, but not expressing heterologous transporters, served as controls. With pathways leading to the intermediates MR or MRG, co‐expression of CcABCC4A2 did not result in increased accumulation of these intermediates relative to the controls (Figure 5). In contrast, when the pathways were reconstructed to include the acylation step, co‐expression of CcABCC4A2 significantly enhanced the accumulation of the acylated intermediates. Specifically, accumulation of the acylated montbretin (Mb) intermediates mini‐Mb, Mb‐XR^2^, and Mb‐R^2^, as well as the complete montbretins, increased by approximately 9‐, 7‐, 7‐, and 4‐fold, respectively. Similar intermediate accumulation patterns were observed by expression of CcABCC4A1 or CcABCC4B, although less pronounced as compared to the expression of CcABCC4A2 (Figure S11, Figure 5).

**FIGURE 5:**
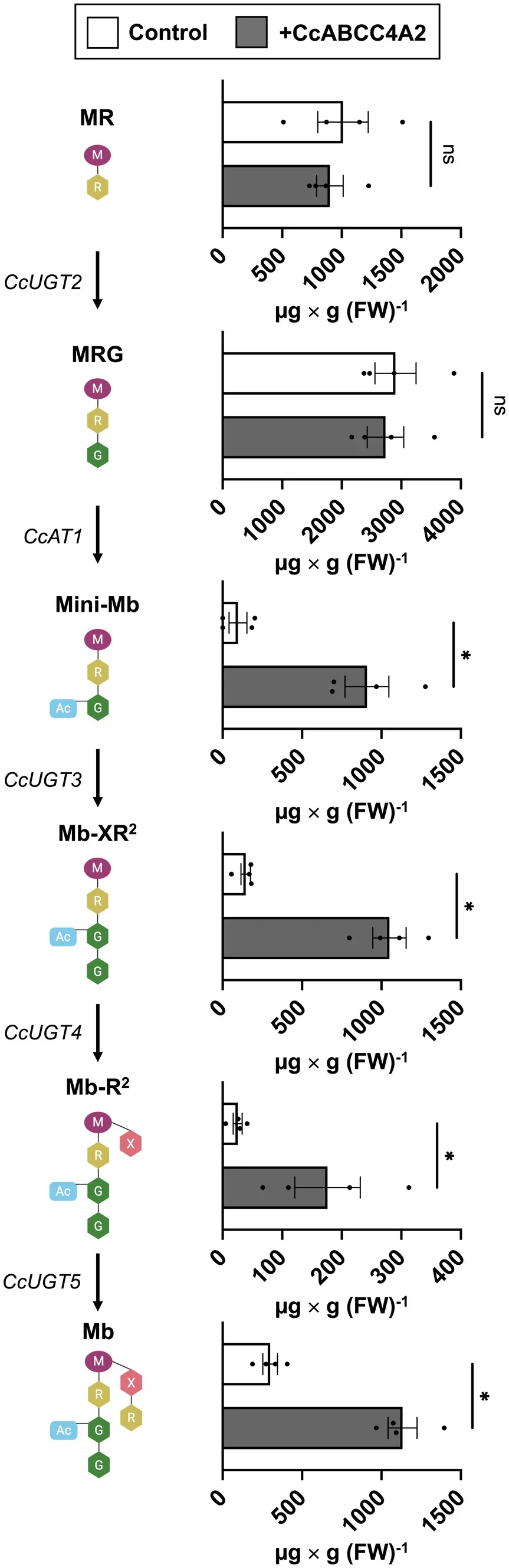
Acylated montbretin biosynthetic intermediates increase in *N. benthamiana* upon expression of CcABCCs. Leaves of *N. benthamiana* transiently expressing genes for respective montbretin intermediate production, with (grey) or without (white) co‐expression of CcABCC4A2 were harvested five days after infiltration. Extracts were analysed using LC–MS and total respective montbretin intermediates were quantified using an external MbA standard curve. For acylated intermediates the abundances of the three different montbretin intermediates were combined. Mean and standard error of four biological replicates (*n* = 4) are plotted, and individual data points are shown as dots. The asterisks indicate a statistically significant difference (*p < 0.05) determined using an unpaired t‐test (Table S7, Data S5). Data of CcABCC4A1 and CcABCC4B shows a similar pattern and is shown in Figure S11. Data of the different acylated montbretin intermediates is shown in Figures S12–S14. Cc, *Crocosmia* × *crocosmiiflora* ; FW, fresh weight; Mb, montbretins; MGC, multi‐gene construct; ns, not significant.

Among the three different montbretins, MbB or the corresponding intermediates towards MbB made up the largest part of total product accumulation compared to MbA and MbC, or the respective intermediates (Figures S12–S14). Furthermore, MbB levels increased with the expression of all three ABCC4 transporters, whereas MbA intermediates only increased significantly upon expression of CcABCC4A2. Specifically, higher levels of mini‐MbA (~38‐fold), MbA‐XR^2^ (~33‐fold), and MbA (~2‐fold) were observed when the corresponding truncated pathways were co‐expressed with CcABCC4A2. In contrast, CcABCC4A1 and CcABCC4B had no or only minor effects on MbA intermediates (Figure S12). Accumulation of MbC intermediates showed a similar pattern to MbA intermediates, with the most pronounced effects again observed for CcABCC4A2 (Figure S14).

### 2.5 CcABCC Homologues From Other Plants Can Enhance Montbretin Accumulation

To assess whether ABCC transporters from other plant species affect montbretin accumulation, we identified and cloned 16 additional ABCC transporters of the ABCC4/14 subclade from montbretia, *Asparagus officinalis, Arabidopsis thaliana, Crocus sativus, Crocus vernus, Iris domestica, Iris pallida, Iris sibirica, N. benthamiana and Zea mays*, in addition to the above‐described three CcABCCs (Data S4, Figure S15). Species were selected to represent members of the Iridaceae family other than montbretia, as well as taxonomically distant species. No MbA, MbB, or MbC, nor any intermediates within the biosynthetic pathway could be detected in methanolic extracts analysed using LC–MS of these plants used in our experiments. The 19 transporters (16 new and the three CcABCC4s) were co‐expressed with the montbretin biosynthetic genes in *N. benthamiana*, and montbretin accumulation was quantified and compared to control plants only expressing the biosynthetic genes (Figure 6, Figure S16). Expression of eight of the 16 ABCCs, specifically CsABCC4B, CvABCC4, IdABCC4, IsABCC4, IpABCC4, IpABCC14, AtABCC14, and NbABCC14, resulted in a ~3‐ to 6‐fold increase in total montbretin content relative to the control. Within the same experiment, expression of CcABCC4A2 led to an ~8‐fold increase in total montbretins. Small to no changes in montbretin accumulation (< 2‐fold) were observed when CcABCC14, CsABCC4A, CvABCC14, AtABCC4, NbABCC4, ZmABCC4, or ZmABCC14 was expressed. Expression of AoABCC14 reduced total montbretin accumulation to trace amounts.

**FIGURE 6:**
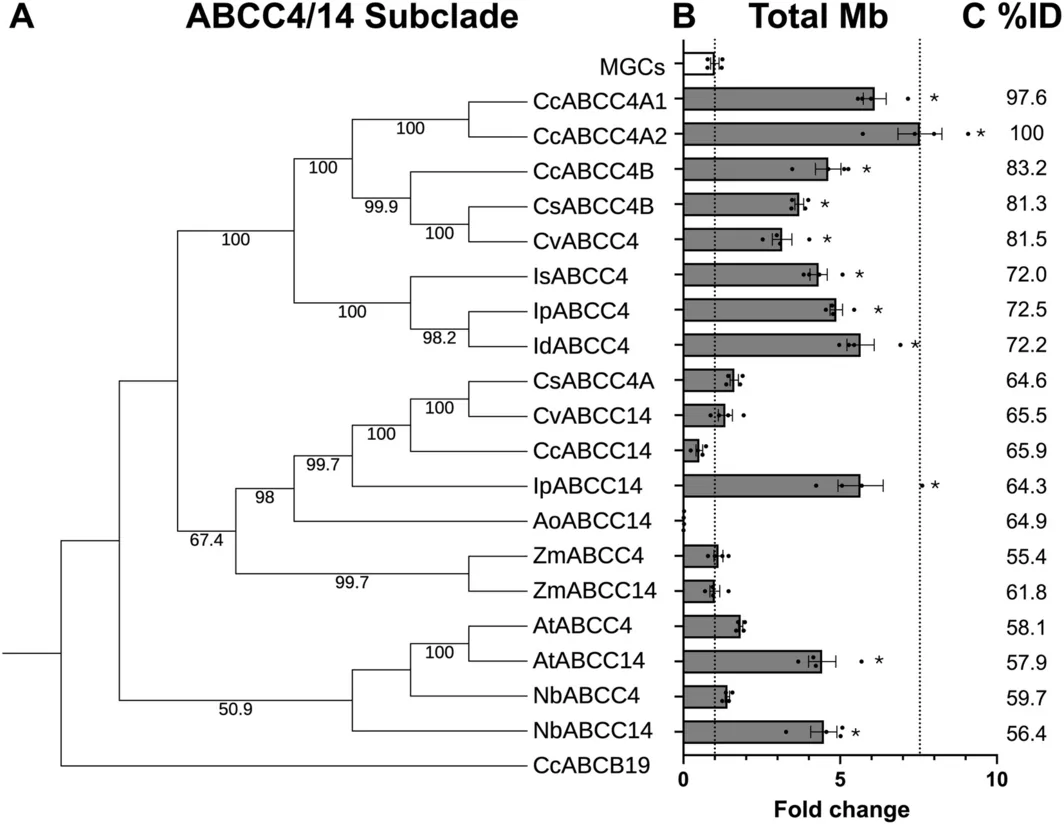
Co‐expression of homologous CcABCC transporters from other plants with montbretin biosynthesis genes increases montbretin accumulation in *N. benthamiana*. (A) Cladogram of the ABCC4/14 transporters from different plant species analysed in this work. The amino acid sequences were aligned using the MUSCLE algorithm in Geneious Prime. The maximum‐likelihood phylogeny was constructed using IQTree and visualized in iTOL. CcABCB19 was used to root the tree. Bootstrap values are shown at each node. (B) Changes of montbretin levels in *N. benthamiana* upon co‐expression of an ABCC transporter. Leaves of *N. benthamiana* transiently expressing montbretin biosynthetic genes with and without co‐expressing a transporter gene were harvested 5 days after infiltration. Extracts were analysed using LC–MS and total montbretin levels (Mb; MbA + MbB + MbC) were quantified using an external MbA standard curve. Montbretin levels are depicted as fold changes relative to levels in plants not co‐expressing a transporter (MGCs), which were set to 1. Data represent the average fold change based on measurements of four biological replicates (*n* = 4). Error bars indicate the SEM. Asterisks indicate a statistically significant difference to the MGC control (one‐way ANOVA followed by Tukey's HSD post hoc test, *p* < 0.05; Table S7, Data S5). Dotted lines indicate the control levels at fold change 1 (left) and the fold change upon co‐expression of CcABCC4A2 (right). (C) Pairwise aa identity (%) of each transporter to CcABCC4A2. *At, Arabidopsis thaliana*; *Ao, Asparagus officinalis*; *Cc, Crocosmia* × *crocosmiiflora* ; *Cs, Crocus sativus*; *Cv, Crocus vernus*; *Id, Iris domestica*; *Ip, Iris pallida*; *Is, Iris sibirica*; *MGC, multi‐gene construct; Mb, montbretins; Nb, Nicotiana benthamiana*; *Zm, Zea mays*.

The effects of the different transporters on individual montbretins (Figure S16) generally mirrored the effects observed for total montbretin accumulation (Figure 6, Figure S16). Mb‐XR^2^ were only detectable in plants expressing the transporters but not in control plants (Figure S17), and Mb‐XR^2^ levels increased upon expression of CcABCC4A2 compared to the MGC control (Figures S3 and S17). Interestingly, expression of CvABCC14, which did not increase montbretin levels, resulted in increased accumulation of Mb‐XR^2^ (~4‐fold; Figure S17) compared to expression of CcABCC4A2. In all other samples, montbretin intermediates were only detected at trace levels.

## 3 Discussion

The plant‐derived specialized metabolite MbA is being developed as a new treatment option for Type 2 diabetes, a disease that is affecting over 800 million people worldwide (Tarling et al. 2008; Williams et al. 2015; Yuen et al. 2016). However, the rare occurrence of MbA in nature, which appears to be limited to montbretia species, and its low natural abundance in these plants poses a considerable supply challenge. Limited natural supply, which is not uncommon for highly valuable plant specialized metabolites (Liu et al. 2024; Martin et al. 2024), may limit drug development and the potential of MbA reaching widespread clinical applications.

However, the MbA biosynthetic pathway has been made tractable for bioengineering, which serves as the foundation to explore scalable MbA production in heterologous systems. In previous work, we elucidated the MbA biosynthetic pathway in montbretia and achieved productions of montbretins, including MbA, in *N. benthamiana* using transient gene expression (Irmisch et al. 2018, 2020; Irmisch, Jancsik, et al. 2019; Irmisch, Ruebsam, et al. 2019; Kruse et al. 2024; Sunstrum et al. 2021). Subsequent optimization of MbA biosynthesis in *N. benthamiana* focused on engineering of precursor supply, specifically of the flavonol myricetin and the phenylpropanoid caffeic acid (Irmisch, Jancsik, et al. 2019; Kruse et al. 2024). Here, we explored whether transport may be another bottleneck and if engineering expression of transporters could be utilized to enhance heterologous MbA production. We identified montbretia CcABC and CcMATE transporters which showed a strong correlation of expression patterns with MbA biosynthetic genes during corm development (Figure 1, Table S1). Expression of CcABCC4A1, CcABCC4A2, CcABCC4B1 or CcMATE18 (Figures 2, 3) significantly increased total montbretin yields in *N. benthamiana* when co‐expressed with the montbretin pathway genes. The tonoplast‐localized CcABCC4A1 and CcABCC4A2 transporters exhibited the most pronounced increase of total montbretins (~6‐ to 7‐fold) and MbA (~2‐fold). Using truncated versions of the montbretin pathway, we showed that the yield‐enhancing effect of CcABCC4 is dependent on the presence of an acyl‐group on the montbretin molecule, suggesting acylation is a transport signal (Figure 5, Figures S11–S14). Furthermore, testing an additional 15 ABCC transporters from other plant species with homology to the three CcABCC4s revealed that the capacity to affect montbretin yield is not limited to plants that naturally produce montbretins (Figure 6), suggesting broader substrate specificity of ABCC transporters. That members of the ABCC transporter family may function with specialized metabolites that they do not encounter in nature, also makes them versatile tools for bioengineering.

### 3.1 Tonoplast‐Localized CcABCC4A1/2 Transporters May Sequester Montbretins Into the Vacuole

Flavonoids are generally synthesized in the cytosol, often at the cytosolic face of the endoplasmic reticulum due to the involvement of cytochrome P450 enzymes, and are subsequently sequestered into the vacuole or exported to the apoplast (Gomez et al. 2009; Falcone Ferreyra et al. 2012; Behrens et al. 2019; Dean et al. 2022). Especially for glycosylated and/or acylated flavonoids, the central vacuole is a common storage compartment, providing both protection of the accumulating metabolites from degradation and protection of the plant cell from potential cytotoxic effects (Gomez et al. 2009; Zhao and Dixon 2010; Martinoia et al. 2012; Zhao 2015; Dean et al. 2022). To cross the tonoplast, active transporter‐mediated movement of flavonoids is necessary due to their hydrophilic nature. A number of studies have implicated ABCC and MATE transporters in vacuolar sequestration of flavonoids (Gomez et al. 2009; Pourcel et al. 2010; Behrens et al. 2019; Kitashova et al. 2024). For example, ABCC1 from *A. thaliana* and *Vitis vinifera* can mediate vacuolar import of anthocyanins and flavonoid glycosides, and the MATE transporter TT12 from *A. thaliana* is required for proanthocyanidin and epicatechin 3′‐*O*‐glucoside accumulation in vacuoles of seed coat cells (Debeaujon et al. 2001; Goodman et al. 2004; Francisco et al. 2013). Based on the effects of transporter expression on MbA yields described here, we hypothesize that MbA is stored in the central vacuole of montbretia corm cells, that this storage involves active transport, and that transporter‐dependent vacuolar sequestration also occurs when MbA is heterologously produced in *N. benthamiana*.

We identified three ABCC4 (CcABCC4A1, CcABCC4A2, and CcABCC4B) and one MATE transporter (CcMATE18) from montbretia which, when individually expressed in montbretin‐producing *N. benthamiana*, increased montbretin levels significantly (Figures 2 and 3). All four transporters were highly expressed in young developing corms—the organs of active montbretin biosynthesis—supporting a role in montbretin transport in montbretia corms (Table S1). It has previously been shown that different transporter classes can facilitate the transport of the same metabolite. For example, transport of anthocyanins such as cyanidin‐3‐*O*‐glucoside can be facilitated by both ABCCs and MATEs (Goodman et al. 2004; Gomez et al. 2009; Zhao and Dixon 2009; Francisco et al. 2013). We propose that CcABCC4A1 and CcABCC4A2 play a key role in vacuolar sequestration of montbretins in montbretia corms. Among the four transporters analysed, CcABCC4A1/A2 exhibited the highest expression levels in developing corms, and their heterologous expression resulted in the greatest increases of montbretin accumulation in *N. benthamiana*. Tonoplast localization of CcABCC4A1 and CcABCC4A2 further supports their role in vacuolar sequestration (Figure 4, Figure S6, Table S5). In contrast, the dual localization of CcMATE18 to both the plasma membrane and tonoplast suggests an additional or alternative function in montbretia (Figure S8, Table S5).

The results of this work demonstrate a yield‐enhancing effect of CcABCC and CcMATE transporters on heterologous montbretin production in *N. benthamiana*. The 7‐fold increase achieved with CcABCC4As is a significant improvement towards the development of *N. benthamiana* as a scalable production system for the antidiabetic MbA. Our results suggest that the effect of CcABCC4s on montbretin yield is due to transport, specifically transporter‐mediated sequestration across the tonoplast into the vacuole. However, an actual transport mechanism and storage of montbretins in the vacuole remain to be proven. It cannot be excluded that the expression of CcABCC4 affects montbretin accumulation by other unknown mechanisms.

### 3.2 Yield Increase Upon CcABCC4 Expression Is Dependent on Metabolite Acylation

Testing substrate specificity of plant specialized metabolite transporters is challenging, due to the lack of suitable expression systems and limited availability of authentic substrates (Chen et al. 2015; Hwang et al. 2016; Nogia and Pati 2021; Banasiak and Jasiński 2022). In this work, we used and advanced an engineered montbretin‐producing *N. benthamiana* system to assess structural features of montbretins that are required for the yield increase observed upon CcABCC4 transporter expression. The system allows for expression of the complete montbretin pathway as well as truncated versions of the pathway leading to the different intermediates in montbretin assembly.

In previous work, we established the CcAT1‐catalysed acylation reaction as the key step in the montbretin assembly pathway (Irmisch et al. 2018). This acylation step produces the first pharmacologically active intermediate, mini‐MbA, with a K_i_ of 400 nM towards HPA (Williams et al. 2015; Tysoe et al. 2019). Furthermore, the acylation step constitutes a major metabolic branchpoint in montbretin biosynthesis. Due to substrate promiscuity of CcAT1, the montbretin pathway diverges into three different routes leading to MbA, MbB, or MbC (Irmisch et al. 2018). As only MbA is a potent HPA inhibitor (K_i_ of 8 nM), diversion of metabolic flux towards MbB and MbC reduces the yield of the desired MbA product. Here, we identified a third important feature of the acylation step in the montbretin assembly pathway. Results from the truncated montbretin pathways in *N. benthamiana* indicate that acylation is a key factor for the montbretin yield‐enhancing effect of CcABCC4, as only acylated montbretin pathway intermediates were increased upon transporter expression (Figure 5, Figures S11–S14). This suggests that acylation may function as a transport signal, which has previously been described for anthocyanins (Gomez et al. 2009; Zhao 2015). If CcABCC4s are indeed involved in vacuolar sequestration of montbretins in montbretia corms, there may be a mechanism that prevents premature sequestration of montbretin pathway intermediates, which may involve effective channelling of intermediates through a metabolon or specific transporter activation (Falcone Ferreyra et al. 2012).

Across the experiments described here, we consistently observed the highest yield increase for MbB or intermediates *en route* to MbB when transporters, especially CcABCC4, were expressed. MbB possesses a coumaroyl moiety instead of the caffeoyl moiety in MbA or the feruloyl moiety in MbC. This predominance of MbB is likely due to *p*‐hydroxycinnamoyl‐CoA precursor availability rather than transporter specificity (Kruse et al. 2024). The prevalence of MbB, which is not an efficient HPA inhibitor, illustrates the need for further pathway engineering to redirect pathway flux towards the bioactive MbA.

### 3.3 ABCC Transporters From Plants Not Known to Produce Montbretins Affect Montbretin Yield in *N. benthamiana*

To date, montbretins have only been described in species of montbretia. Thus, we wanted to assess whether the montbretin yield‐enhancing effect is specific to the expression of CcABCC4 from montbretia or a more general feature of plant ABCCs, as ABCCs are known to possess broad substrate specificity (Banasiak and Jasiński 2022). To this end, we tested 15 ABCC4/14‐clade transporters from nine plant species that do not produce montbretins, along with montbretia ABCC14, for their ability to increase montbretin levels upon co‐expression in *N. benthamiana*. Eight transporters within the ABCC4/14 subclade increased montbretin levels, but neither sequence similarity nor their position within the phylogeny was indicative of this activity (Figure 6). Homologous transporters from different plant species have been shown to recognize similar substrates, despite differences in species origin and metabolite profiles (Yu and De Luca 2013; Demessie et al. 2017; Banasiak and Jasiński 2022). Interestingly, the here tested AtABCC14 has previously been shown to transport acylated anthocyanins which are structurally similar to montbretins (Dean et al. 2022), providing an explanation of why co‐expression of AtABCC14 increased montbretin levels. This supports the idea that ABC transporters often recognize more general molecular features rather than specific individual compounds (Hwang et al. 2016; Lefèvre and Boutry 2018).

Co‐expression of ABCC14 from *N. benthamiana* also increased montbretin levels (Figure 6). This result suggests that NbABCC14 may mediate baseline montbretin transport in the montbretin‐producing *N. benthamiana*. However, the increase in total montbretin and MbA accumulation upon NbABCC14 overexpression indicates that endogenous expression of this transporter was a bottleneck. Nevertheless, metabolic flux may still be limited by the absence of specialized transport mechanisms or insufficient transporter expression. Consistent with this, co‐expression of CcABCC4A2 resulted in higher montbretin accumulation compared to co‐expression of NbABCC14 (Figure 6). These findings suggest that, although ABCC transporters can exhibit substrate promiscuity, they might still have been evolutionarily optimized for the efficient transport of their endogenous ligands. Similar observations have been reported for tropane alkaloid biosynthesis in engineered yeast (Srinivasan and Smolke 2020, 2021). Transporters from the high tropane alkaloid‐producing species *Atropa belladonna* supported substantially greater accumulation of scopolamine and hyoscyamine than homologues from *Nicotiana tabacum* , where these compounds accumulate only at relatively low levels.

### 3.4 MbA Production in Transient *N. benthamiana* Is a Viable Option

Agrobacterium‐mediated transient expression in *N. benthamiana* is amenable to scale‐up. It is used to rapidly produce recombinant proteins without the need for stable genomic alterations (Ward et al. 2021). In addition, preparative production of heterologous plant specialized metabolites has been achieved (Reed et al. 2017). Challenges might be posed by increasing pathway complexity. This can impose additional metabolic burden on the host system and requires co‐infiltration of multiple *Agrobacterium* cultures possibly associated with lower production titers (Forestier et al. 2021). For the latter, we have previously developed a multigene construct system, reducing the number of independently delivered constructs required for transient MbA production (Kruse et al. 2024). Including CcABCC4A2 within these multigene constructs will ensure a low number of co‐infiltrated constructs to reduce process complexity and improve reproducibility.

Our transient MbA production system produces about 0.25–0.40 mg MbA × g (FW)^−1^ which corresponds to about 38–62 mg MbA per plant per production cycle, based on reported *N. benthamiana* leaf biomass yields (~155 g FW per plant under molecular farming conditions) (Hou et al. 2022). A single *C. crocosmiiflora* “Red King” plant produces about 75 mg MbA in 1 year, assuming about 3 mg MbA × g^−1^ FW within their corms and 5 corms per plant weighing 5 g each (Irmisch et al. 2018). Montbretia grows annually and to our knowledge, no agricultural production system has been established. In contrast, vertical farming systems for *N. benthamiana* exist where year‐round, highly intensified production cycles are possible. Thus, using transient *N. benthamiana* plants for MbA production already offers greater annual production capacity per unit growing area. It has to be noted that for all calculations on MbA yield, absolute titers and biomass yields can vary between batches and production facilities depending on plant growth and physiological conditions.

In parallel, the knowledge gained from optimization of the transient *N. benthamiana* system will inform the development of stable heterologous production systems in plants, as well as alternative production hosts such as yeast.

## 4 Conclusion

Our work illustrates the potential of transporters as bioengineering tools to enhance metabolite yield in engineered heterologous systems. By expressing CcABCC4A2 in montbretin‐producing *N. benthamiana*, we increased montbretin yield 7‐fold and the yield of the anti‐diabetic MbA 2‐fold, indicating that transport and subcellular compartmentation constrained metabolite production. The yield of 2.5 mg × g (FW)^−1^ montbretins from *N. benthamiana* highlights the theoretical potential of *N. benthamiana* as an MbA production platform. However, the higher abundance of MbB compared to MbA underscores the need for further engineering to improve caffeoyl‐CoA availability and flux towards MbA.

The observation that transporters from species not known to produce montbretins can increase montbretin levels demonstrates that engineering tools can be acquired from a broad variety of plant species, exploiting the broader substrate specificity of transporters. Ultimately, a deeper understanding of transporter substrate recognition is needed to enable more rational selection or engineering of transporters as tools in engineered metabolite production systems.

## 5 Materials and Methods

### 5.1 Plant Material

*C.* × *crocosmiiflora* “Red King” corms were provided by Kwekerij Davelaar (The Netherlands), *C. sativus* and *C. vernus* corms were purchased from Bol.com (The Netherlands), *I. domestica* rhizomes were kindly gifted by Dr. Rogier van Vugt and Dr. Roderick Bouman (Hortus Botanicus Leiden, The Netherlands) and *Z. mays* “Sweet Nugget” seeds (Chrestensen, Erfurt, Germany) were gifted by Dr. Tobias Koellner (Max Planck Institute for Chemical Ecology, Jena, Germany). All plants were grown in climate chambers with a photoperiod of 16 h light and 8 h dark, at 70% relative humidity and 23°C. Young corms or rhizome sections were harvested approximately 4 months after planting and whole *Z. mays* plants were harvested 2 weeks after seed germination. *I. pallida and I. siberica* plants were purchased from Kwekerij Meeuwissen Voorhout (The Netherlands) and rhizome sections were harvested. Flowers and leaves of 6‐week‐old *A. thaliana* Col‐0 grown from seeds at 23°C were provided by Sofia Stiegert (Institute of Biology Leiden, The Netherlands). Green *A. officinalis* stalks were purchased at Albert Heijn (The Netherlands). Leaves of 4‐week‐old *N. benthamiana* plants (grown as stated above, from seed) were used for infiltration, RNA isolation and/or metabolite extraction. After harvesting, all plant material was flash‐frozen in liquid nitrogen, ground to a fine powder and stored at −80°C until further use. For each plant species, 100 mg (fresh weight, FW) material was extracted with 1 mL of LC‐grade 50% MeOH (v/v) by sonication (pulse setting, 15 min, RT). Extracts were stored at −20°C until LC–MS analysis (up to max. 1 month).

### 5.2 Identification of Candidate Transporter Sequences and Gene Amplification

For the identification of putative transporter sequences from montbretia (*C*. × *crocosmiiflora*), a BLASTP search using ABC and MATE transporter sequences from existing literature (Data S1 and S2) was conducted against the translated transcriptome database that covers a time course of montbretia corm development (Irmisch et al. 2020) as well as a database of young and old corms (Irmisch et al. 2018). Duplicates were removed and sequences ≥ 350 aa length were selected. A reciprocal BLASTP search against the non‐redundant protein database (NCBI) was carried out and only sequences returning the terms ABC, MATE, MRP (multidrug resistance protein) or DTX (protein DETOXIFICATION) were kept (Verrier et al. 2008). Aa sequences were manually assessed to remove redundancies (cut‐off: 97% pairwise identity for CcABCs and 99% for CcMATEs). On the nucleotide level, shorter contigs were combined into predicted full‐length sequences whenever possible through manual editing. This yielded 76 putative CcABC and 30 putative CcMATE sequences. The ABCI subfamily was not included in this work due to their atypical structure (often prokaryotic‐type ABC subunits), highly diverse short sequences and unknown association with specialized metabolite transport (Banasiak and Jasiński 2022).

To construct phylogenies, the predicted transporter aa sequences were aligned with those from other plant species (for the full list of transporters see Data S1 and S2) using the MUSCLE 5.1 plugin (PPP algorithm; 0 HMM perturbations, no guide tree permutations, 4 threads) in Geneious Prime 2025.1.2 (https://www.geneious.com). This was done individually for CcABC (File S1), CcABCC (File S2), CcMATE (File S3), ABCC (File S4), and ABCC4/14 (File S5) transporters following the construction of maximum‐likelihood phylogenetic trees using IQtree (best model selection: JTT; 1000 ultrafast bootstraps; Trifinopoulos et al. 2016), and iTOL v6 (Letunic and Bork 2024) for visualization. Subfamilies were identified based on clustering with known transporters from the literature. CcABCCs were chosen as primary candidates due to their described tonoplastic localization (Kang et al. 2011; De Brito Francisco and Martinoia 2018; Demurtas et al. 2019). CcABCBs and CcABCGs were included as secondary candidates. CcMATE and CcABC transporter candidates were narrowed down based on their expression patterns in the time course dataset (Irmisch et al. 2020). Only CcMATEs and CcABCs showing ≥ 5‐ or ≥ 2‐fold higher expression at the June 10th compared to the October 6th time point, respectively, were kept (Table S1). Final candidates were selected based on their correlation of expression (Pearson's correlation of ρ ≥ 0.9 or ≥ 0.8 for CcMATEs and CcABCs, respectively) with MbA biosynthetic genes (*CcUGT1‐5, CcAT1*) (Irmisch et al. 2018, 2020; Irmisch, Jancsik, et al. 2019; Irmisch, Ruebsam, et al. 2019). Heatmaps were constructed using TBTools‐II (Chen et al. 2023) to visualize expression patterns for CcABCs and CcMATEs separately.

To identify transporter homologues from *A. officinalis, A. thaliana, C. sativus, I. domestica, I. pallida, I* *. sibirica* , *N. benthamiana*, and *Z. mays*, CcABCC4s were used as a query in a BLASTP search against translated transcriptomic or genomic data available in online data repositories (Data S3). The top BLASTP hit for each plant was selected for cloning.

To amplify the complete ORFs of the putative transporters, RNA was isolated and converted into cDNA using the HiScript II 1st Strand cDNA Synthesis Kit (Vazyme, Nanjing). For young *C.* × *crocosmiiflora* “Red King” and *Crocus* corms, *Iris* rhizomes, and *Asparagus* stalks, a previously described RNA isolation protocol was used (Kolosova et al. 2004; Irmisch et al. 2018). The E.Z.N.A. Plant RNA Kit (Omega Bio‐Tek, Norcross) was used for isolation of RNA from leaves of *A. thaliana* and *N. benthamiana*, and whole *Z. mays* plants. ORFs were amplified from cDNA using Phanta Max Polymerase (Vazyme, Nanjing) and cloned into the pJET1.2/Blunt vector (Thermo Fisher Scientific). Sequences were verified using Sanger (*CcMATEs*) or Oxford Nanopore whole plasmid sequencing (*CcABCs*) (Eurofins Genomics Europe, Germany). A list of all cloned transporters and oligonucleotide sequences used for their amplification can be found in Data S4 and Table S8. *CvABCC14* and *CvABCC4* were amplified using oligonucleotide primers designed for *CsABCC4A* and *CcABCC4B*, respectively.

### 5.3 Plasmid Assembly and Transporter Characterization in *N. benthamiana*

Generation of plasmids and subsequent agroinfiltration of *N. benthamiana* leaves was performed as described previously (Irmisch et al. 2018). Briefly, the complete ORF for each transporter was cloned into the pCAMBIA230035Su (pCAM) vector using the USER fusion cloning method (Nour‐Eldin et al. 2006; Geu‐Flores et al. 2007) followed by heat‐shock transformation of individual plasmids into the *Agrobacterium tumefaciens* strain C58pMP90 (GV3101pMP90) (Koncz and Schell 1986). Additionally, previously described pCAM vectors harbouring the individual MbA biosynthetic genes (35S_pro_:{gene} with ORFs encoding {*CcUGT1‐5* or *CcAT1* or *CcFLS* or *CcCYP2* or *CcMYB4* or *CcHCT*}), pBIN::*p19*, and multigene constructs (MGCs) were used for transformation (Irmisch et al. 2018, 2020; Irmisch, Jancsik, et al. 2019; Irmisch, Ruebsam, et al. 2019; Kruse et al. 2024). MGC1 carries [35S_pro_:{*CcUGT1, CcUGT2, CcUGT3, CcUGT4, CcUGT5*, and *CcAT1*}] and MGC2 harbours [35S_pro_:{*CcFLS, CcCYP2, CcMYB4, CcHCT*, and *P19*}] (Kruse et al. 2024). For USER cloning, primers were designed based on sequences described in Table S8, with 5′‐GGCTTAAU‐3′ and 5′‐GGTTTAAU‐3′ overhangs on forward and reverse primers, respectively.

One milliliter of an overnight *A. tumefaciens* culture was used to inoculate 50 mL cultures (LB medium containing 50 μg × L^−1^ kanamycin, 25 μg × L^−1^ rifampicin and 25 μg × L^−1^ gentamycin) which were then grown overnight at 28°C, 220 rpm. Cells were harvested by centrifugation and resuspended in infiltration buffer (10 mM MES, 10 mM MgCl_2_, 100 μM acetosyringone, pH 5.6) to a final OD_600_ of 0.5 and incubated for 3 h at RT, shaking at 220 rpm. Before infiltration, equal volumes of each strain required for a particular combination of genes were combined and used to infiltrate the abaxial side of the 3rd and 4th youngest leaves of 3 to 4‐week‐old *N. benthamiana* plants using a needleless syringe. After 5 d, one leaf disc was harvested for each of the two infiltrated leaves and pooled, weighed, shock‐frozen in liquid nitrogen, and stored at −80°C until extraction. 100 mg (fresh weight, FW) leaf material was extracted with 1 mL of LC‐grade 50% MeOH (v/v) by shaking at 120 rpm for 2 h at RT. Extracts were stored at −20°C until LC–MS analysis.

For studying the effect of different transporters on MbA accumulation, MGCs were used to achieve MbA production while pathway reconstruction experiments were carried out with individual pCAM plasmids. Constructs and combinations used for the different infiltration experiments are summarized in Tables S6 and S9. The results represent data from at least four independent biological replicates per infiltration combination, and experiments were performed at least twice independently.

### 5.4 Subcellular Localization of Transporter Candidates in *N. benthamiana*

DeepLoc2.1 and TargetP‐2.0 were used to predict the subcellular location and signal peptide sequences within the translated amino acid sequences of MbA assembly genes (CcUGT1‐5, AT1), and CcABCC4s and CcMATE28 (Almagro Armenteros et al. 2019; Nielsen 2025). To visualize the subcellular location of CcABCC and CcMATE transporters upon transient expression in *N. benthamiana* leaves, pCAM plasmids harbouring the CcABCC4 ORFs connected to a GFP at their C‐terminus or the CcMATE ORFs attached to a GFP at their N‐terminus were constructed. A proline‐glycine‐proline polypeptide linker was used to fuse the transporters to GFP. To generate a tonoplast marker, mRuby2 was fused to the C‐terminus of the *A. thaliana* gamma‐tonoplast intrinsic protein (γTIP, amplified from cDNA of *A. thaliana* flowers as described for transporters) by a glycine‐serine linker and cloned into pCAM. *N. benthamiana* infiltration was performed as described above. Transporter–GFP constructs were co‐infiltrated with p19 and, where indicated, with pCAM:γTIP::mRuby2 as a tonoplast marker. The styryl dye FM4‐64 (Invitrogen) was used as a plasma membrane marker and infiltrated into transformed plants immediately prior to imaging (5 mg × L^−1^ in infiltration buffer) in samples not co‐expressing pCAM:γTIP::mRuby2. A detailed list of used constructs and infiltration combinations can be found in Tables S6 and S9. Imaging of the abaxial side of infiltrated leaves was performed 5 days post‐infiltration. All images were acquired using a Nikon AXE confocal microscope (20X magnification, pinhole:1 AU, GFP ex: 488 nm, em: 510 to 530 nm; γTIP‐mRuby2 and FM4‐64 ex: 561 nm, em: 600 to 620 nm) and processed and analysed using Fiji (v. 2.16.0). Co‐localization was assessed using Van Steensel's analysis within Fiji, and Pearson correlation coefficients (r) were calculated between the two fluorescence channels. Data reflect the mean of four measurements, two cells of each of two biological replicates.

### 5.5 Metabolite Extraction and LC–MS Analysis

For LC–MS analysis, plant extracts were diluted 1:10 in 50% (v/v) LC‐grade MeOH containing 20 μg × mL^−1^ of the internal standard umbelliferone (Sigma‐Aldrich). Samples were subjected to liquid chromatography (LC) performed on a Shimadzu Nexera X2 UHPLC (Shimadzu, Tokyo, Japan) equipped with a Waters Acquity HSS C18 column (1.8 μm, 100 Å, 2.1 × 100 mm; Waters) using water (mobile phase A) and acetonitrile (mobile phase B) both acidified with 0.1% (v/v) formic acid. The flow rate was 0.5 mL × min^−1^ at a column temperature of 30°C. The elution profile was: 0 to 1 min, 5% B; 1 to 2 min, 5% to 17% B; 2 to 10 min, 17% to 20% B; 10 to 11 min, 20% to 100% B (held at 100% B for 4 min). The LC was coupled to a Shimadzu 9030 Q‐TOF mass spectrometer (MS) equipped with an electrospray operated in negative ionization mode (interface voltage, −3.0 kV; temperature, 300°C; nebulizing gas flow, 3 L × min^−1^; drying gas flow 10 L × min^−1^). Full scan MS spectra (*m*/*z* 100–1700, scan rate 10 Hz, ID enabled) were followed by two data‐dependent MS/MS spectra (*m*/*z* 100–1700, scan rate 10 Hz, ID disabled) for the two most intense ions per scan. Ions over an intensity threshold of 1500 were isolated at the tuning file Q1 resolution, fragmented using collision‐induced dissociation (fixed collision energy, ce 20 eV), and excluded for 1 s before being re‐selected for fragmentation. Data processing was conducted using MZmine 4 (Schmid et al. 2023), and visualized and analysed in Graphpad Prism (v.10). An authentic standard of myricetin was purchased from TCI (Tokyo, Japan) and MR was retrieved from Cayman Chemical Company (Ann Arbor, MI, USA). MRG (Irmisch et al. 2018), Mini‐MbA, MbA‐XR^2^, MbA‐R^2^ and MbA (Williams et al. 2015) were available from previous research. Montbretin and montbretin intermediate yields were quantified based on an external MbA standard curve.

## Author Contributions

S.I. conceived the research, S.I. and C.H. designed and supervised the research, C.H. carried out the experimental work, S.I, C.H., F.C.C., L.H.K. and J.B. and analysed the data and interpreted the results; S.I. and C.H. wrote the paper; all authors read, edited, and approved the final version of the manuscript.

## Conflicts of Interest

The authors declare no conflicts of interest.

## Supporting information


**Figure S1:** Cladogram of the montbretia ABC transporter family expressed in corms.
**Figure S2:** Cladogram of the montbretia MATE transporter family expressed in corms.
**Figure S3:** Mb‐XR^2^ produced by engineered *N. benthamiana* expressing CcABCC4 transporters.
**Figure S4:** Co‐expression of CcABCB and CcABCG transporters does not affect montbretin accumulation in *N. benthamiana*.
**Figure S5:** Cytosolic localization of GFP in *N. benthamiana* leaves.
**Figure S6:** CcABCC4A1 localizes to the tonoplast in *N. benthamiana* leaves.
**Figure S7:** CcABCC4B localizes to the tonoplast in *N. benthamiana* leaves.
**Figure S8:** CcMATE18 localizes to the tonoplast and the plasma membrane in *N. benthamiana* leaves.
**Figure S9:** Montbretin accumulation in *N. benthamiana* co‐expressing GFP‐tagged CcABCC4 transporters.
**Figure S10:** Montbretin accumulation in *N. benthamiana* co‐expressing the GFP‐tagged CcMATE18 transporter.
**Figure S11:** Montbretin and montbretin intermediate accumulation in *N. benthamiana* co‐expressing CcABCCs.
**Figure S12:** MbA and MbA intermediate accumulation in *N. benthamiana* co‐expressing CcABCCs.
**Figure S13:** MbB and MbB intermediate accumulation in *N. benthamiana* co‐expressing CcABCCs.
**Figure S14:** MbC and MbC intermediate accumulation in *N. benthamiana* co‐expressing CcABCCs.
**Figure S15:** Cladogram of ABCC transporters from montbretia and other plants.
**Figure S16:** Changes of montbretin levels in *N. benthamiana* upon co‐expression of an ABCC transporter with montbretin biosynthetic genes.
**Figure S17:** Changes of Mb‐XR^2^ levels in *N. benthamiana* upon co‐expression of an ABCC transporter.

**Table S1:** Differential expression of ABCC and MATE transporter transcripts in montbretia corms over time.
**Table S2:** Montbretin levels produced in engineered *N. benthamiana*.
**Table S3:** Predicted subcellular locations of MbA biosynthesis genes and of ABCC and MATE transporters.
**Table S4:** N‐terminal presequence prediction using TargetP‐2.0 of MbA biosynthesis genes and of ABCC and MATE transporters.
**Table S5:** Colocalization analysis (Pearson) of CcABCC4s and CcMATE18 in *N. benthamiana*.
**Table S6:** Infiltration combinations used per experiment.
**Table S7:** Statistical output of all montbretin (intermediate) quantifications.
**Table S8:** Oligonucleotides used in this study.
**Table S9:** Constructs used for agroinfiltration in this work.

**Data S1:** ABC amino acid sequences from literature and those from *Crocosmia* × *crocosmiiflora* identified in this work.
**Data S2:** MATE amino acid sequences from literature and those from *Crocosmia* × *crocosmiiflora* identified in this work.
**Data S3:** Sequence list of selected ABCC transporters.
**Data S4:** Sequence list of all cloned genes in this work.
**Data S5:** Detailed statistics output for the main figures.

**File S1:** Amino acid alignment of CcABC transporters used for construction of the phylogeny shown in Figure S1.
**File S2:** Amino acid alignment of CcABCC transporters used for construction of the phylogeny shown in Figure 1A.
**File S3:** Amino acid alignment of CcMATE transporters used for construction of the phylogeny shown in Figure S2.
**File S4:** Amino acid alignment of ABCC transporters used for construction of the phylogeny shown in Figure S15.
**File S5:** Amino acid alignment of ABCC4/14 transporters used for construction of the phylogeny shown in Figure 6A.

## Data Availability

The montbretia transporter sequences characterized in this study have been deposited in NCBI GenBank with the following accession numbers: *CcABCC4A1*, PZ577546; *CcABCC4A2*, PZ577547; *CcABCC4B*, PZ577548; *CcMATE18*, PZ577549. Other sequences can be found in Data S4. Transcriptome data from *Crocosmia* × *crocosmiiflora* has been published previously in the NCBI/GenBank under the project number PRJNA389589 (SRP108844).
