# Supplementary material for: Tonoplast‐Localized ABCC Transporters Enhance Heterologous Production of the Antidiabetic Montbretin A and Its Analogues in Nicotiana benthamiana
Source: Plant Biotechnol J. 2026 Sep 28;24(11):5991–6006. doi: 10.1111/pbi.70765 (PMC13618170; doi:10.1111/pbi.70765)
Supplement: Supplementary file 1 [file PBI-24-5991-s003.pdf]

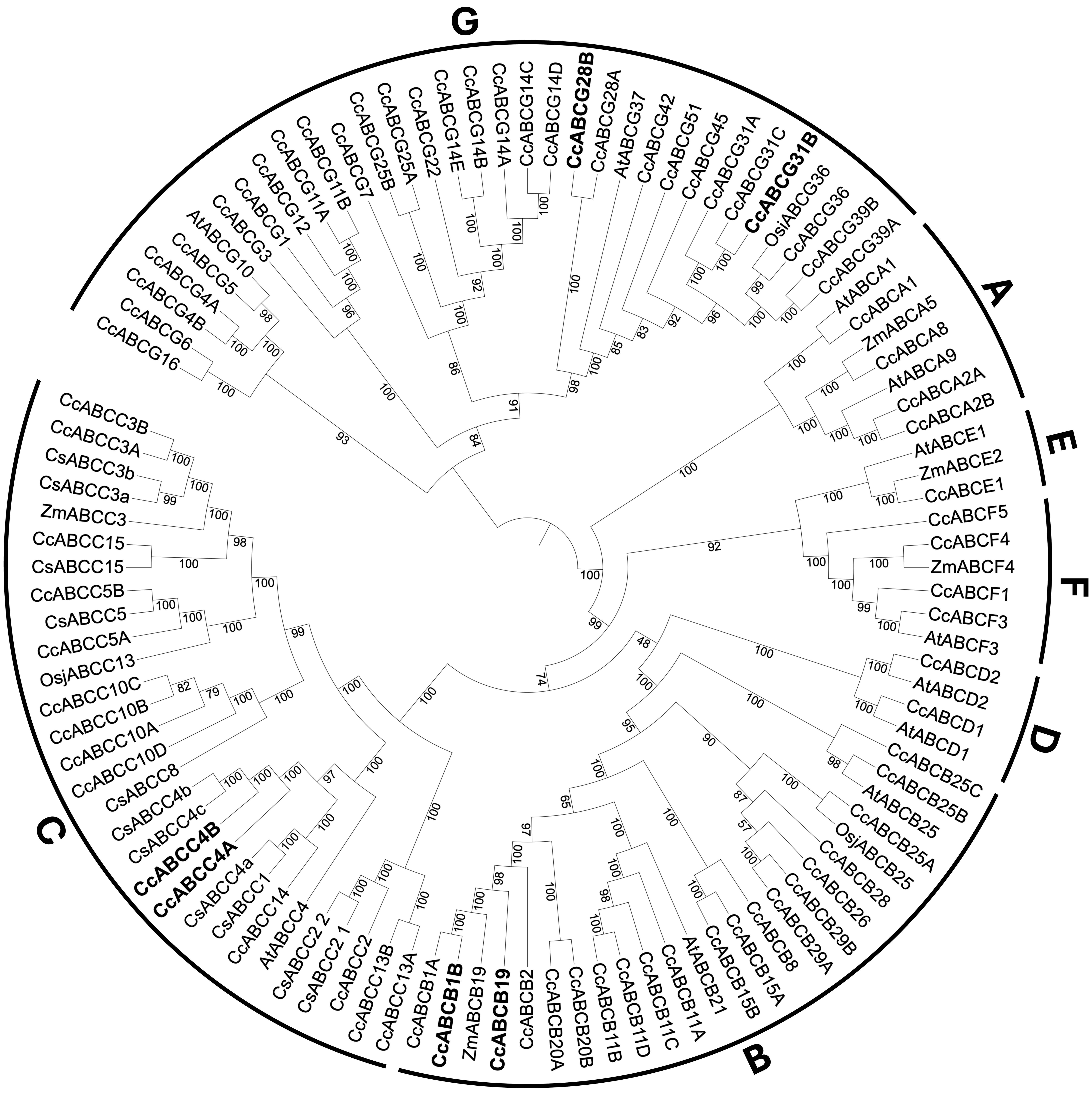

Figure S1. Cladogram of the montbretia ABC transporter family expressed in corms. Predicted amino acid sequences of montbretia ABC transporters were identified from a corm transcriptome and were aligned with ABC transporters from other plant species using the MUSCLE algorithm in Geneious Prime. The maximum-likelihood tree was constructed using IQTree and visualized using iTOL. All transporters were manually annotated and a reciprocal BLASTP against a nonredundant protein database (NCBI) was used to identify and name montbretia homologs based on their closest relatives. Subfamilies are labeled with their respective letters on the outside of the cladogram. The phylogeny was rooted at midpoint. Bootstrap values (ultrafast) are shown at each respective node. ABC transporters characterized in this work are in bold. Amino acid sequences and accession numbers for each sequence can be found in Data S1. *At: Arabidopsis thaliana; Cc: Crocosmia x crocosmiiflora; Cs: Crocus sativus; Cv: Crocus vernus; Osi/j: Oryza sativa indica/japonica; Zm: Zea mays.*

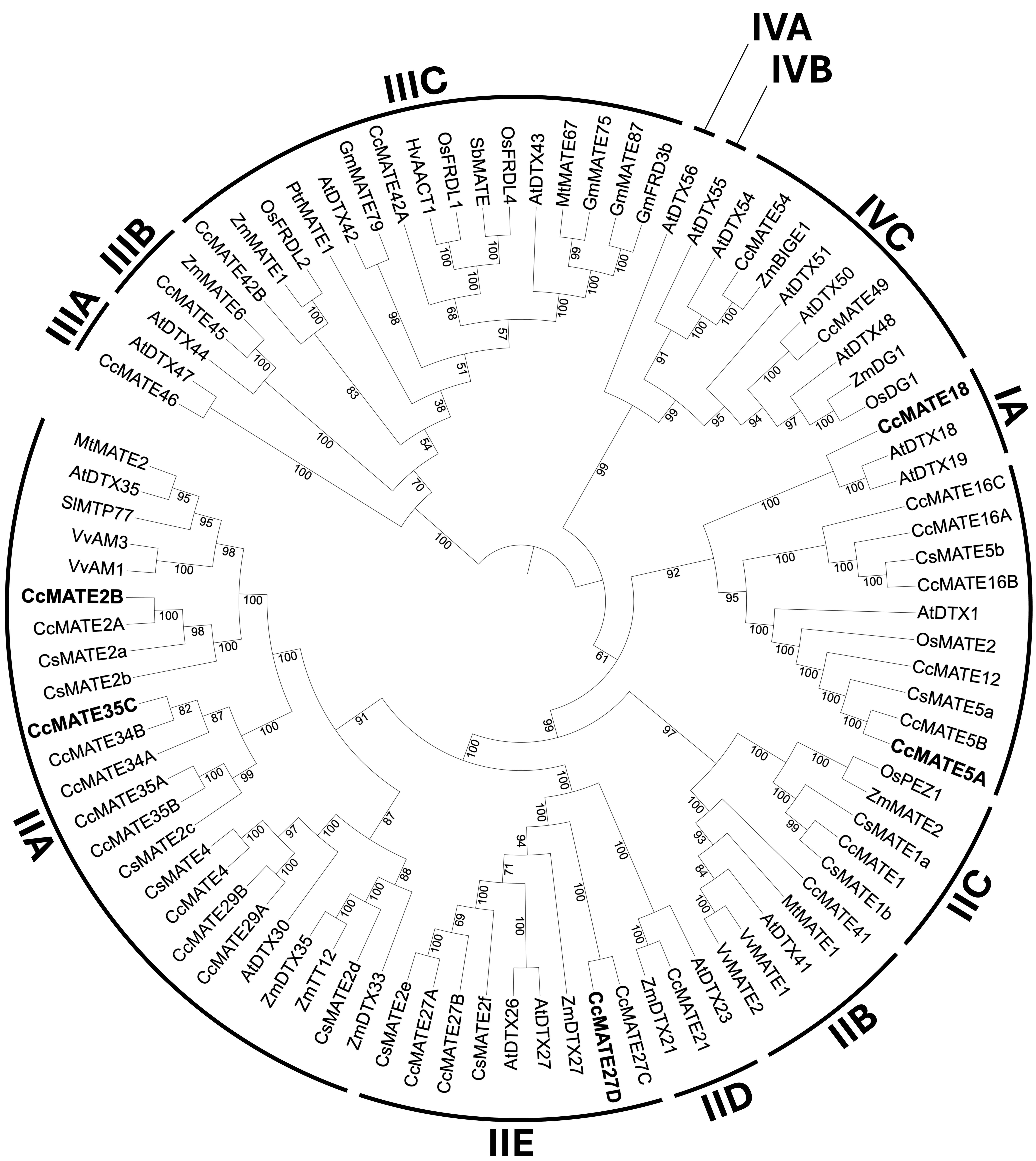

Figure S2. Cladogram of the montbretia MATE transporter family expressed in corms. Predicted amino acid sequences of montbretia MATE transporters were identified using a corm development transcriptome and aligned with MATE transporters from other plant species using the MUSCLE algorithm in Geneious Prime. A maximum-likelihood tree was constructed using IQTree and visualized using iTOL. All transporters were manually annotated and a reciprocal BLASTP against a nonredundant protein database (NCBI) was used to identify and name montbretia homologs based on their closest relatives. Subfamilies are labeled with their respective letters on the outside of the tree. The phylogeny was rooted at midpoint. Bootstrap values (ultrafast) are shown at each respective node. MATE transporters characterized in this work are in bold. Accession numbers for each sequence can be found in Data S2. *At: Arabidopsis thaliana; Cc: Crocosmia x crocosmiiflora; Cs: Crocus sativus; Cv: Crocus vernus; Gm: Glycine max ; Gs: Glycine soja; Hv: Hordeum vulgare; Mt: Medicago truncatula; Os: Oryza sativa; Ptr: Populus trichocarpa; Sb: Sorghum bicolor: Sl: Solanum lycopersicum Vv: Vitis vinifera; Zm: Zea mays.*

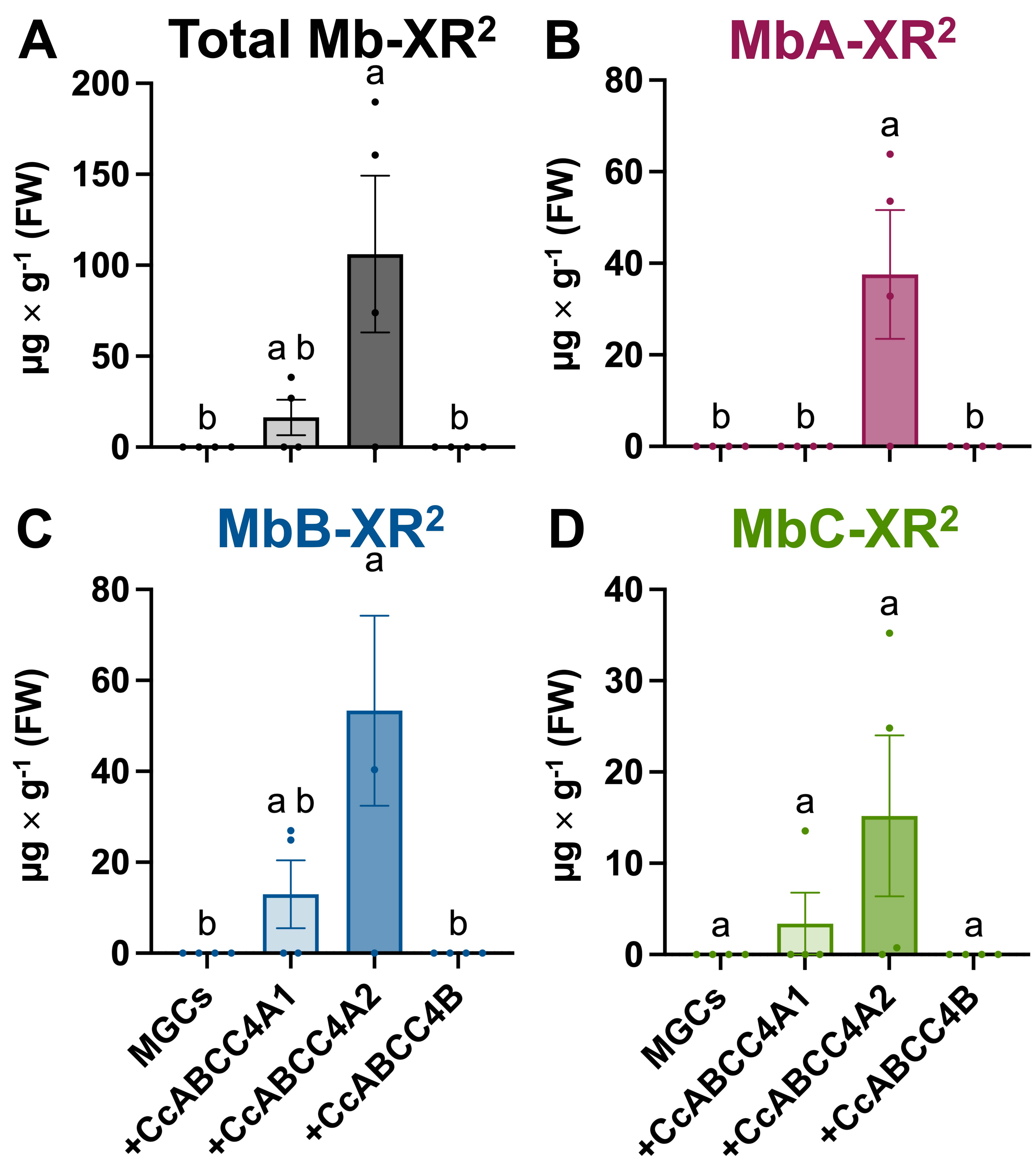

Figure S3. Mb-XR² produced by engineered *N. benthamiana* expressing CcABCC4 transporters. Leaves of *N. benthamiana* transiently expressing Mb biosynthesis genes with and without co-expressing a transporter gene were harvested five days after infiltration. Extracts were analyzed using LC-MS and total Mb-XR² (MbA-XR²+MbB-XR²+MbC-XR²; **A**), MbA-XR² (**B**), MbB-XR² (**C**) and MbC-XR² (**D**) levels were quantified using an external MbA standard curve. Mean and standard error of four biological replicates ($n = 4$) are plotted, and individual data points are shown as dots. Different letters indicate statistically significant differences between groups (one-way ANOVA followed by Tukey's HSD post hoc test, $P < 0.05$). Mb, montbretin; FW, fresh weight.

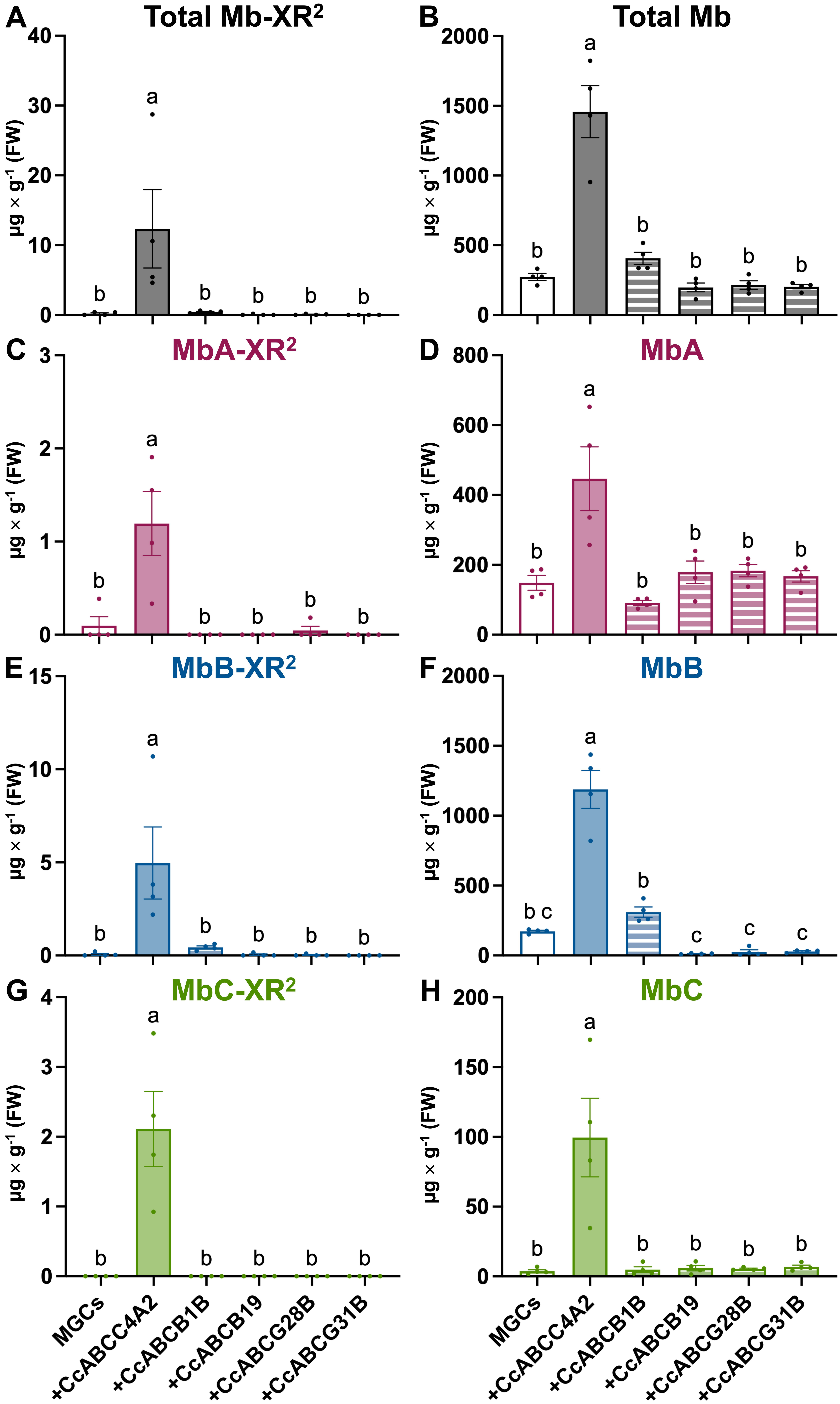

Figure S4. Co-expression of CcABCB and CcABCG transporters does not affect montbretin accumulation in *N. benthamiana*. Leaves of *N. benthamiana* transiently expressing Mb biosynthesis genes with and without co-expressing a transporter gene were harvested five days after infiltration. Extracts were analyzed using LC-MS and total Mb-XR^2 (MbA-XR^2+MbB-XR^2+MbC-XR^2; **A**), MbA-XR^2 (**C**), MbB-XR^2 (**E**) and MbC-XR^2 (**G**) and total Mb (MbA+MbB+MbC; **B**), MbA (**D**), MbB (**F**) and MbC (**H**) levels were quantified using an external MbA standard curve. Mean and standard error of four biological replicates ($n = 4$) are plotted, and individual data points are shown as dots. Different letters indicate statistically significant differences between groups (one-way ANOVA followed by Tukey's HSD post hoc test, $P < 0.05$). Mb, montbretin; FW, fresh weight.

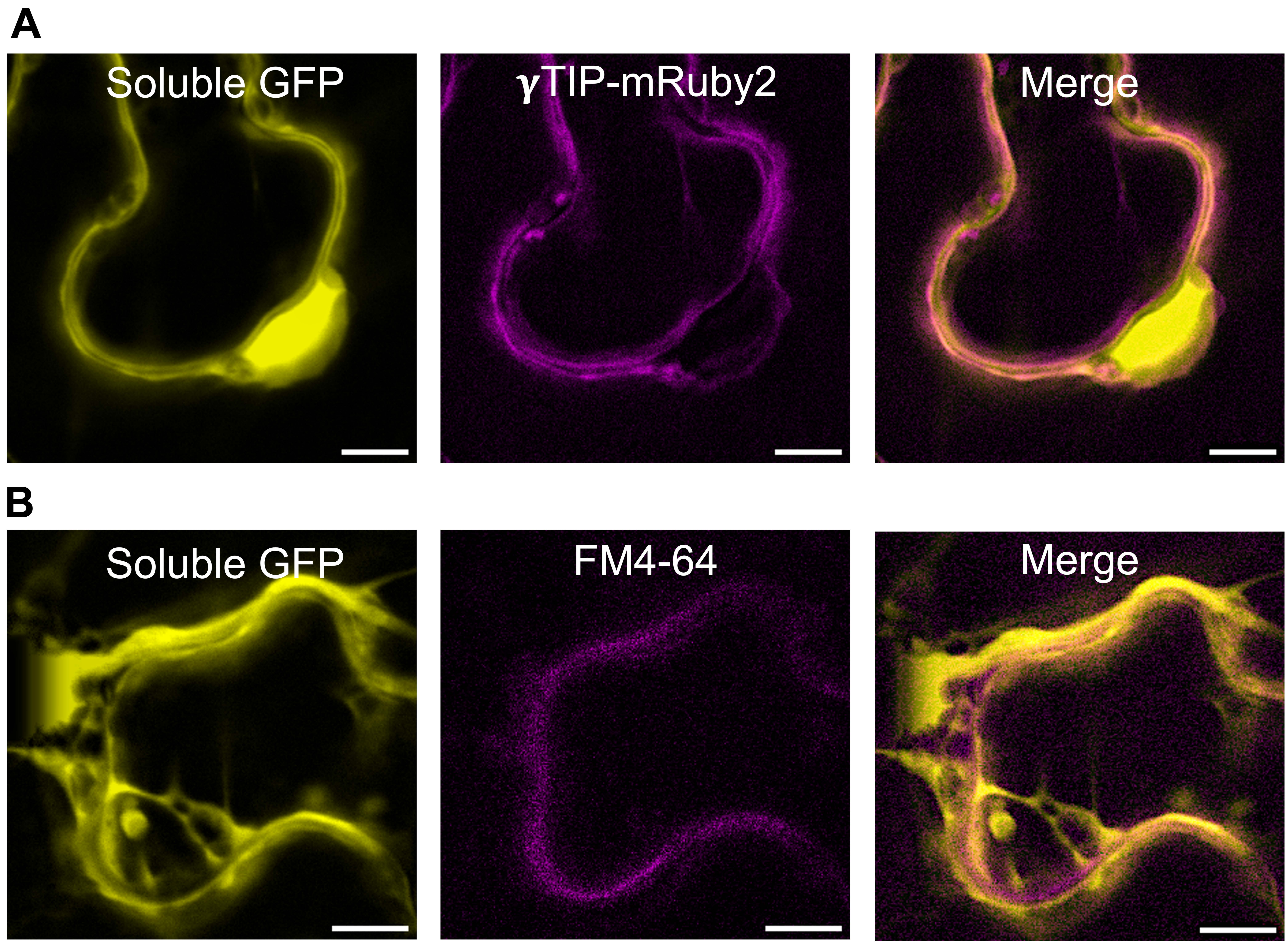

Figure S5. Cytosolic localization of GFP in *N. benthamiana* leaves. GFP was transiently expressed in *N. benthamiana*. Leaf tissue was subjected to confocal microscopy five days after infiltration to visualize the cytosol. The tonoplast marker AtγTIP::mRuby2 was co-infiltrated with GFP (**A**) and FM4-64 was used to stain the plasma membrane of plants expressing GFP (**B**). Representative images of at least three biological replicates are shown. Scalebars measure 10 µm.

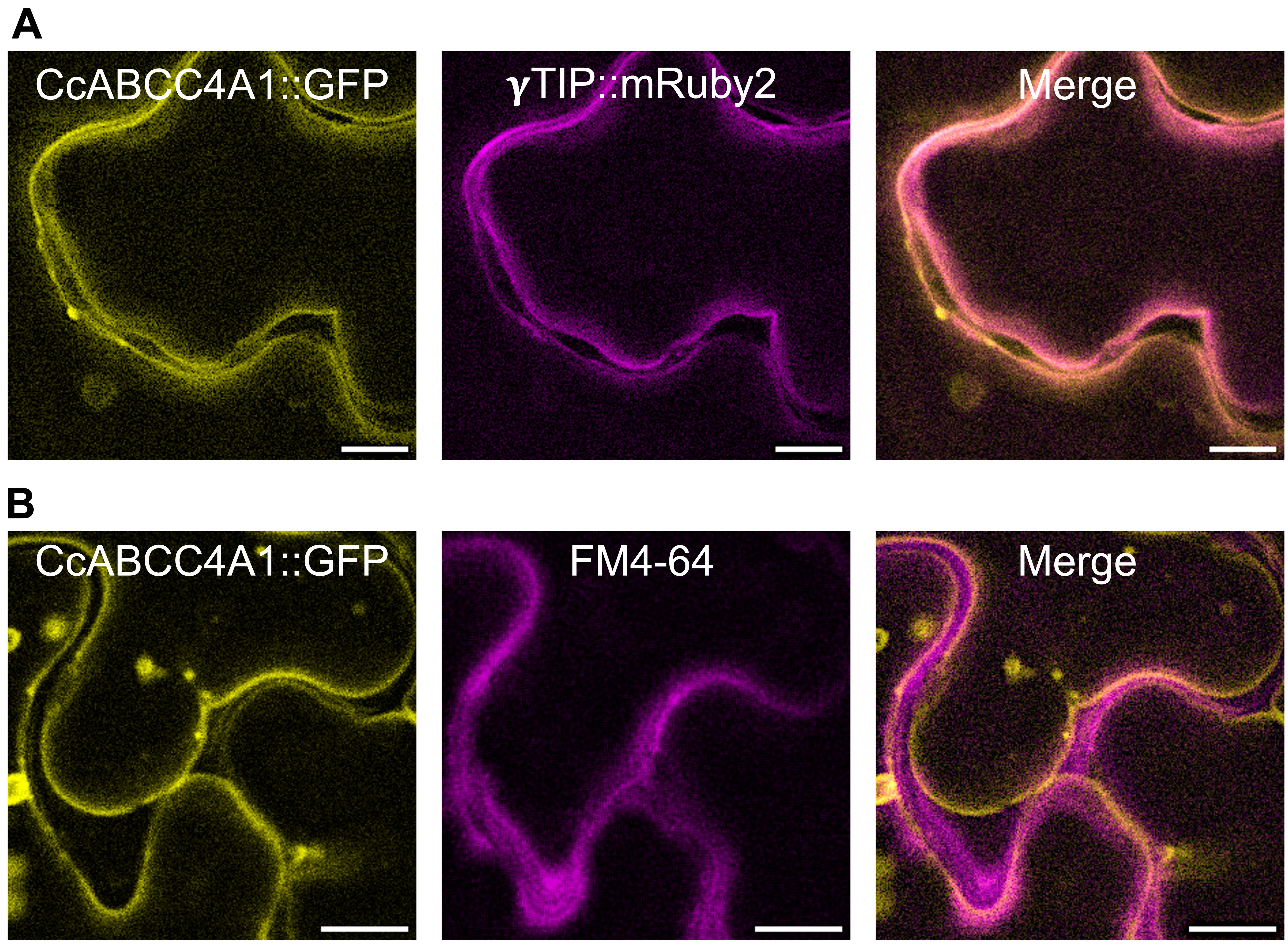

Figure S6. CcABCC4A1 localizes to the tonoplast in *N. benthamiana* leaves. GFP was fused to the C-terminus of CcABCC4A1 and transiently expressed in *N. benthamiana*. Leaf tissue was subjected to confocal microscopy five days after infiltration to visualize the subcellular transporter localization. The tonoplast marker AtγTIP::mRuby2 was co-infiltrated with CcABCC4A1::GFP (**A**) and FM4-64 was used to stain the plasma membrane of plants expressing CcABCC4A1::GFP (**B**). Representative images of at least three biological replicates are shown. Scalebars measure 10 µm.

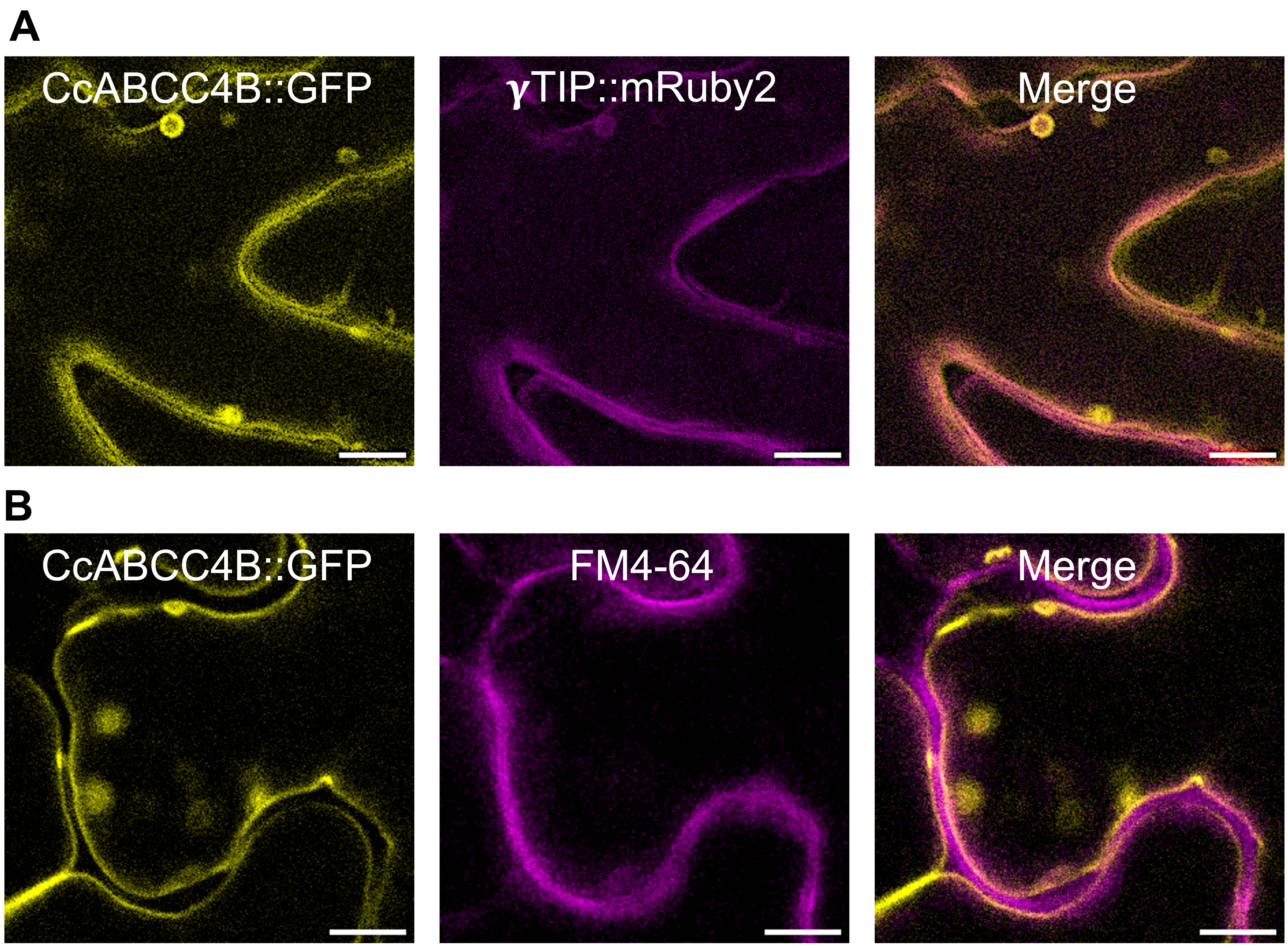

Figure S7. CcABCC4B localizes to the tonoplast in *N. benthamiana* leaves. GFP was fused to the C-terminus of CcABCC4B and transiently expressed in *N. benthamiana*. Leaf tissue was subjected to confocal microscopy five days after infiltration to visualize the subcellular transporter localization. The tonoplast marker AtγTIP::mRuby2 was co-infiltrated with CcABCC4B::GFP (**A**) and FM4-64 was used to stain the plasma membrane of plants expressing CcABCC4B::GFP (**B**). Representative images of at least three biological replicates are shown. Scalebars measure 10 µm.

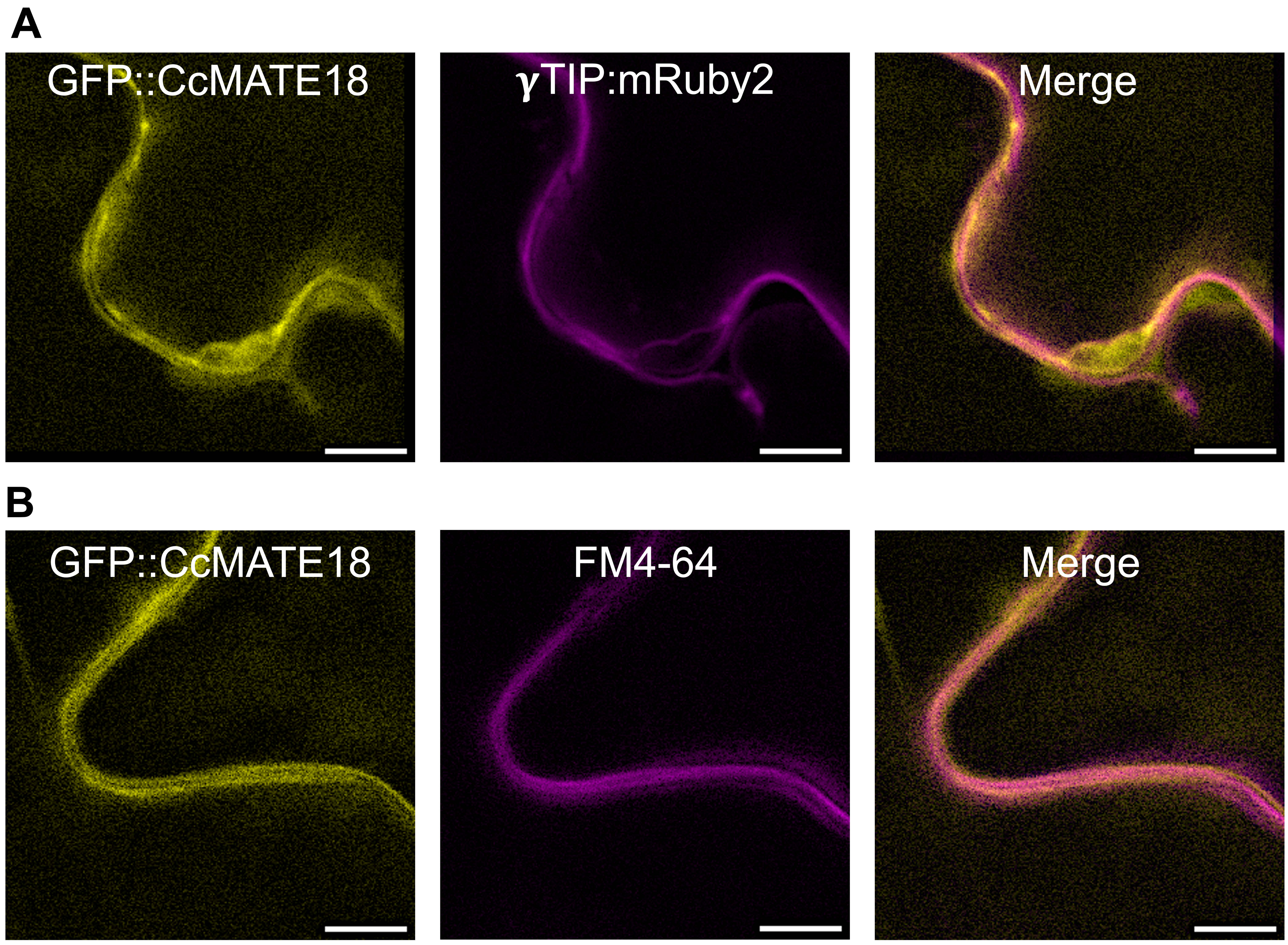

Figure S8. CcMATE18 localizes to the tonoplast and the plasma membrane in *N. benthamiana* leaves. GFP was fused to the N-terminus of CcMATE18 and transiently expressed in *N. benthamiana*. Leaf tissue was subjected to confocal microscopy five days after infiltration to visualize the subcellular transporter localization. The tonoplast marker AtγTIP::mRuby2 was co-infiltrated with GFP::CcMATE18 (**A**) and FM4-64 was used to stain the plasma membrane of plants expressing GFP::CcMATE18 (**B**). Representative images of at least three biological replicates are shown. Scalebars measure 10 µm.

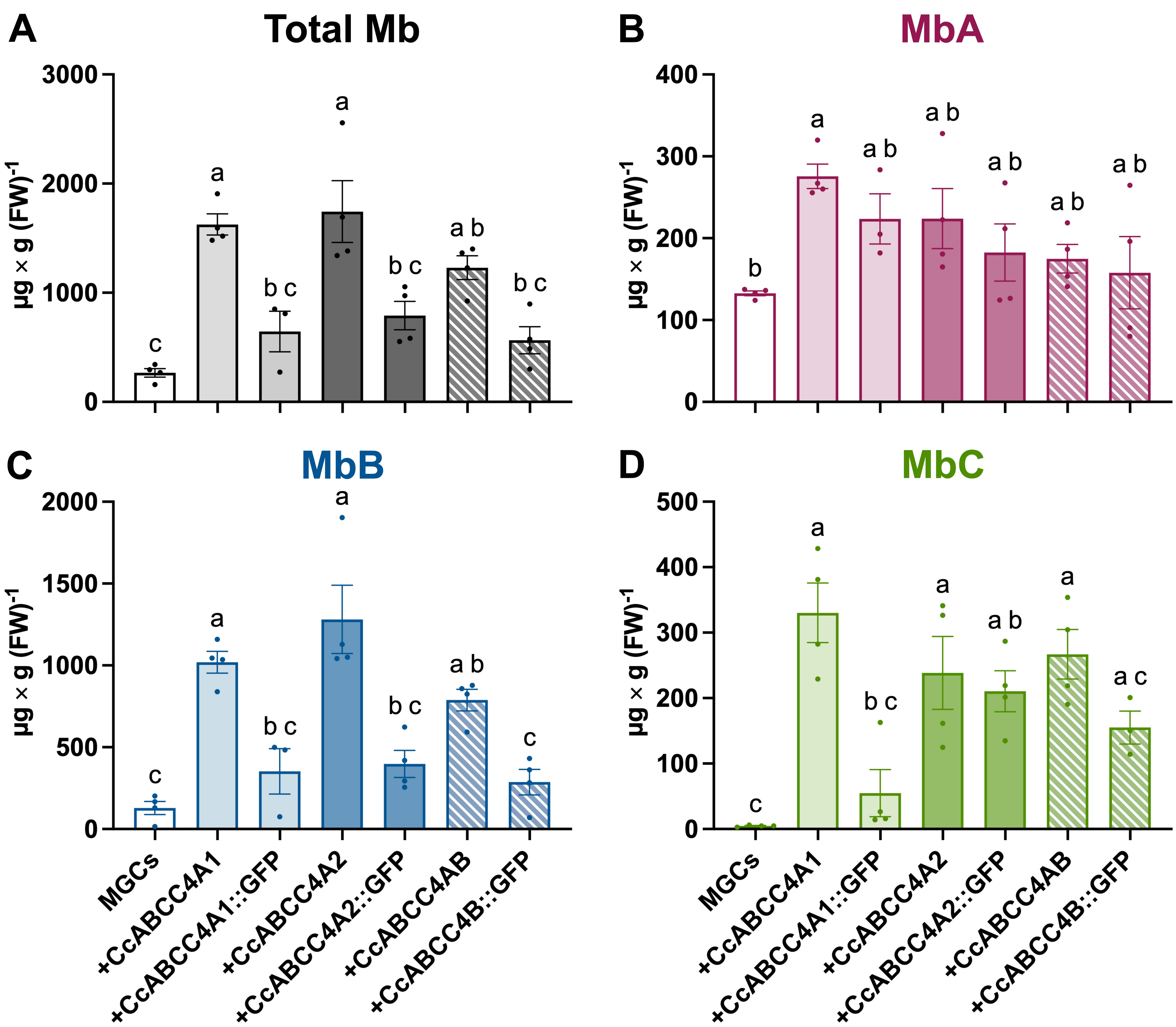

Figure S9. Montbretin accumulation in *N. benthamiana* co-expressing GFP-tagged CcABCC4 transporters. Leaves of *N. benthamiana* transiently expressing Mb biosynthesis genes with and without co-expressing a GFP-tagged or untagged transporter gene were harvested five days after infiltration. Extracts were analyzed using LC-MS and total Mb (MbA+MbB+MbC; **B**), MbA (**C**), MbB (**D**) and MbC (**E**) levels were quantified using an external MbA standard curve. Mean and standard error of four biological replicates (n = 4) are plotted, and individual data points are shown as dots. Different letters indicate statistically significant differences between groups (one-way ANOVA followed by Tukey's HSD post hoc test, $P < 0.05$). Mb, montbretin; FW, fresh weight.

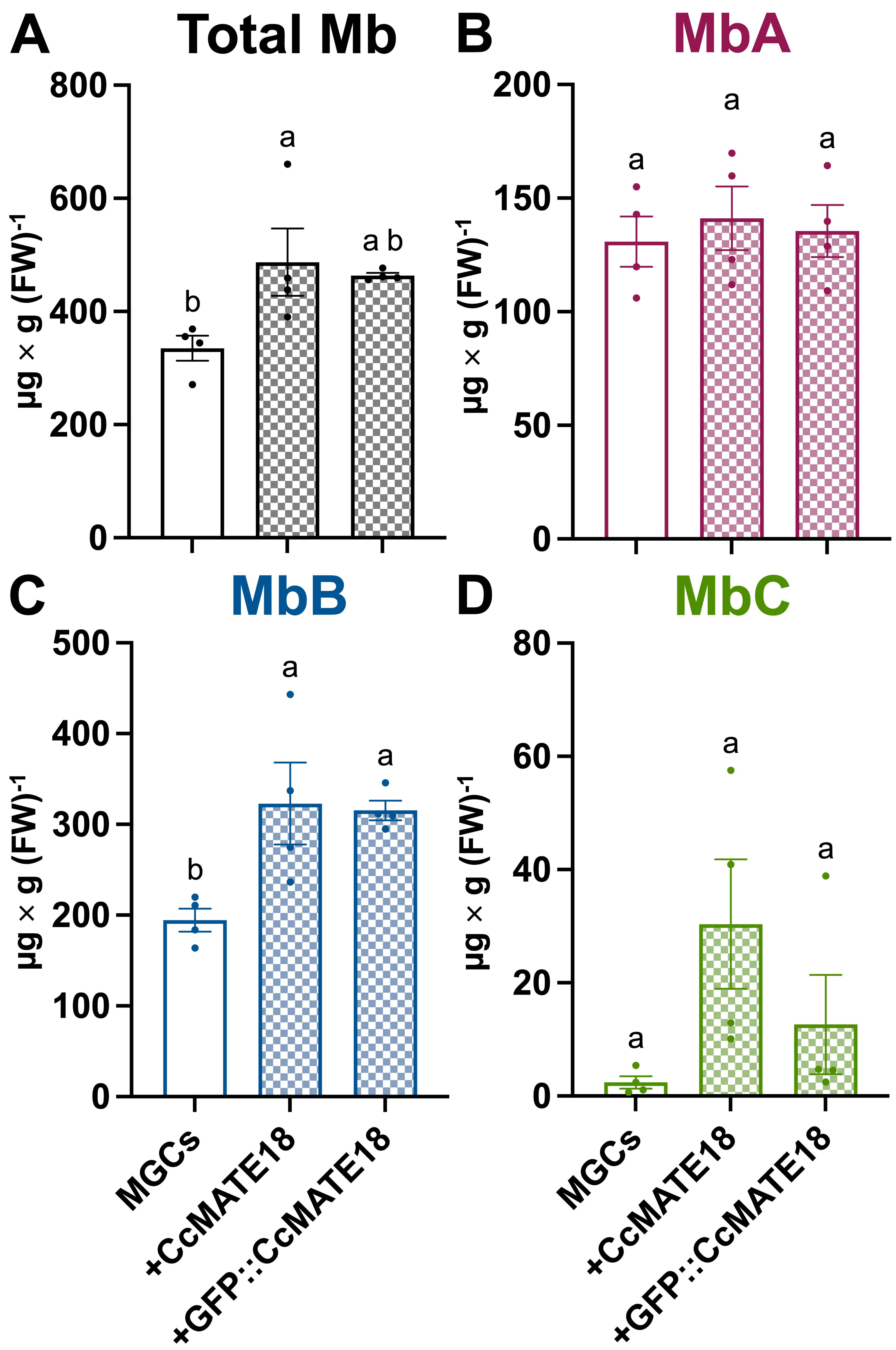

Figure S10. Montbretin accumulation in *N. benthamiana* co-expressing the GFP-tagged CcMATE18 transporter. Leaves of *N. benthamiana* transiently expressing the Mb biosynthesis genes with and without co-expressing transporter genes were harvested five days after infiltration. Extracts were analyzed using LC-MS and total Mb (MbA+MbB+MbC; **A**), MbA (**B**), MbB (**C**) and MbC (**D**) levels were quantified using an external MbA standard curve. Mean and standard error of four biological replicates ($n = 4$) are plotted, and individual data points are shown as dots. Different letters indicate statistically significant differences between groups (one-way ANOVA followed by Tukey's HSD post hoc test, $P < 0.05$). Mb, montbretin; FW, fresh weight.

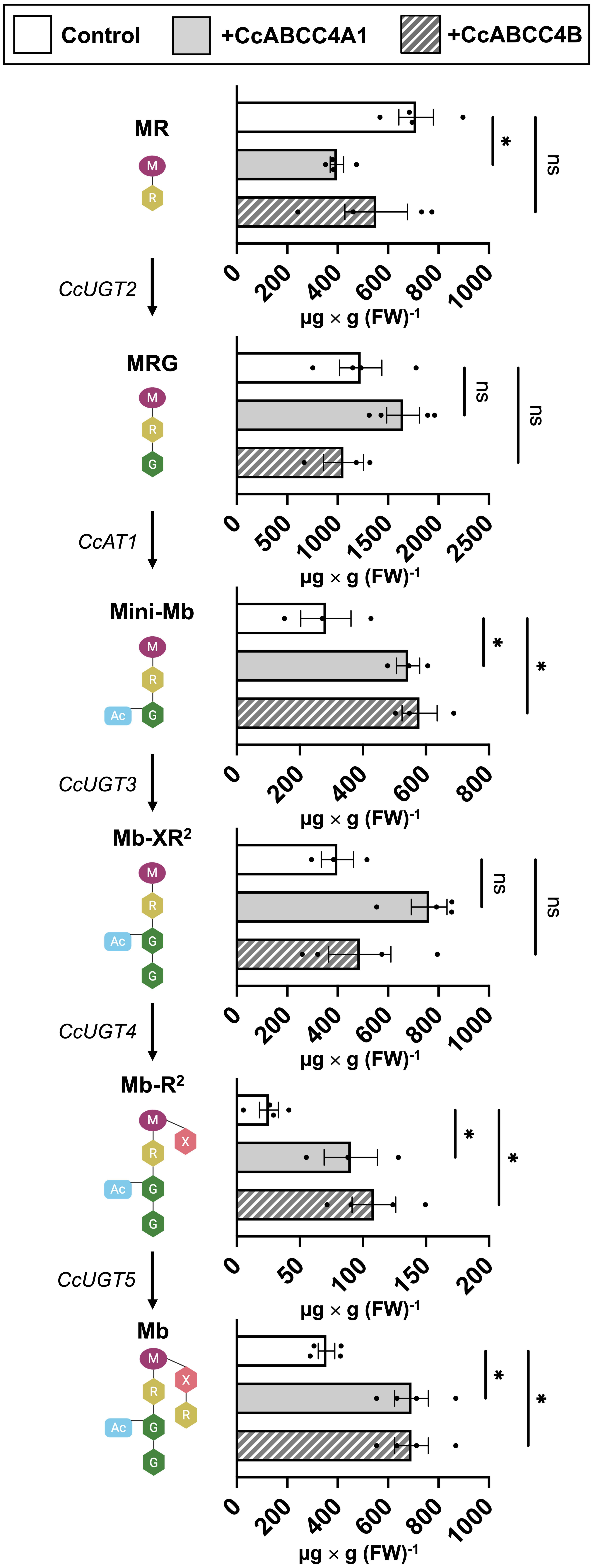

Figure S11. Montbretin and montbretin intermediate accumulation in *N. benthamiana* co-expressing CcABCCs. Leaves of *N. benthamiana* transiently expressing genes for respective Mb intermediate production, with CcABCC4A1 (light grey), CcABCC4B (grey hatched), or without (white) transporter co-expression were harvested five days after infiltration. Extracts were analyzed using LC-MS and total respective montbretin intermediates were quantified using an external MbA standard curve. For acylated intermediates the three different montbretin intermediates were combined. Mean and standard error of four biological replicates (n = 4) are plotted, and individual data points are shown as dots. The asterisks indicate a statistically significant difference (*P <0.05) as determined using an unpaired t-test. Data for the different acylated Mb intermediates is shown in Figures S12, S13, and S14. FW, fresh weight; Mb, montbretins; Cc, *Crocosmia* x *Crocosmiiflora;* M, myricetin; R, rhamnose; G, glucose; Ac, acyl; X, xylose; ns, not significant.

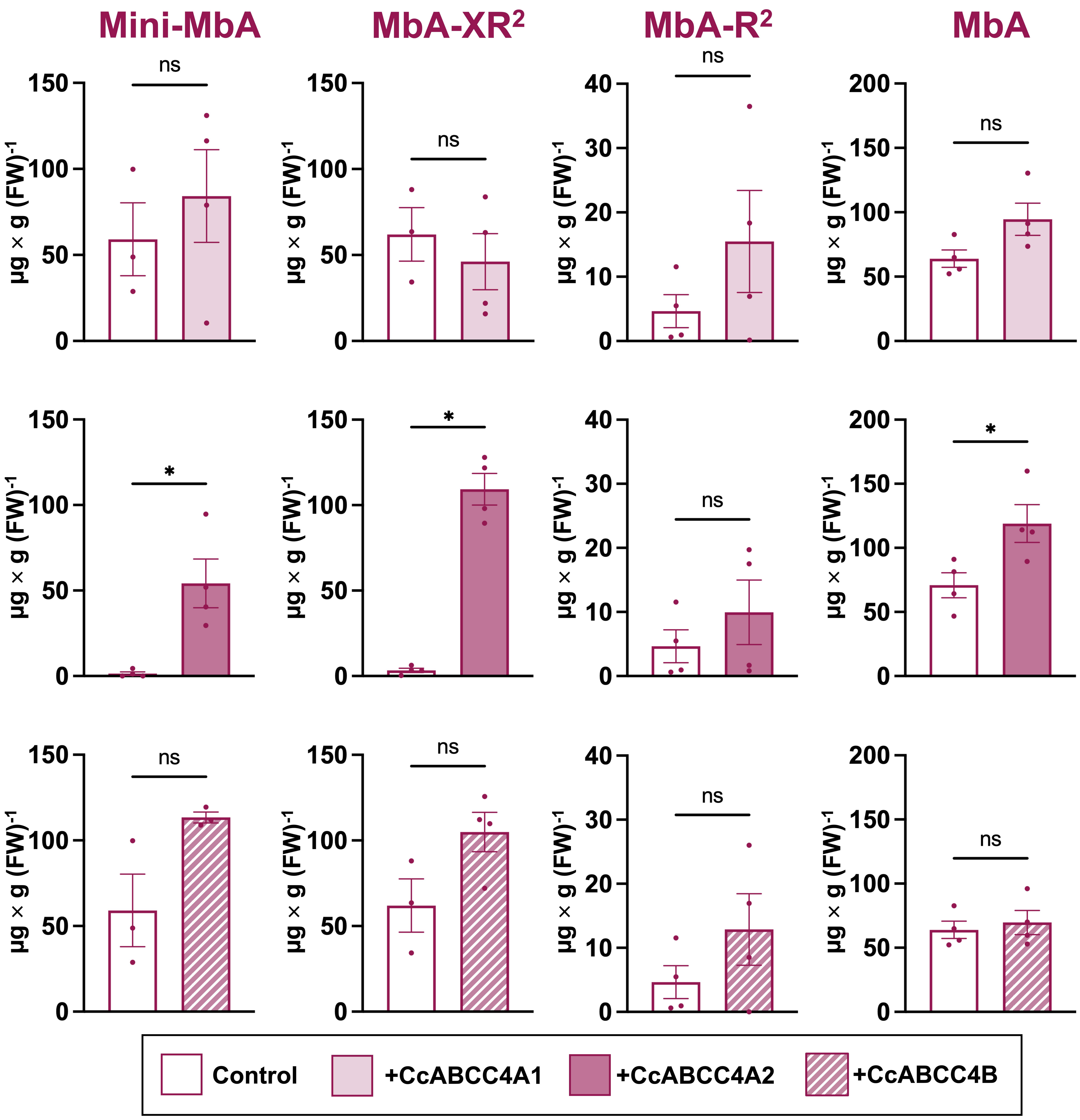

Figure S12. MbA and MbA intermediate accumulation in *N. benthamiana* co-expressing CcABCCs. Leaves of *N. benthamiana* transiently expressing genes for respective Mb intermediate production, with CcABCC4A1 (light pink), CcABCC4A2 (dark pink), CcABCC4B (pink hatched), or without (white) transporter co-expression were harvested five days after infiltration. Extracts were analyzed using LC-MS and MbA intermediates were quantified using an external MbA standard curve. Mean and standard error of four biological replicates (n = 4) are plotted, and individual data points are shown as dots. The asterisks indicate a statistically significant difference (*P <0.05) as determined using an unpaired t-test. FW, fresh weight; ns, not significant.

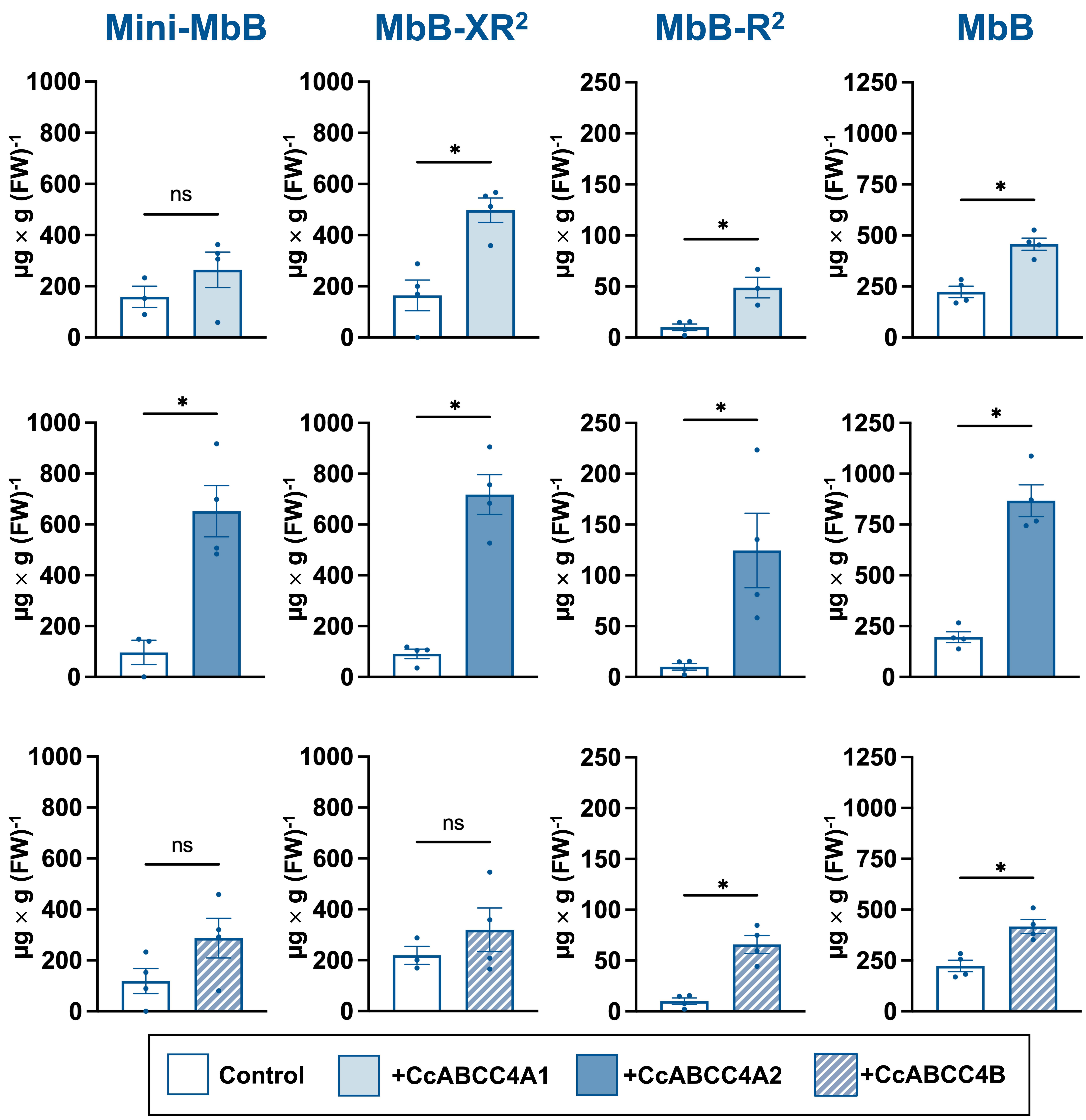

Figure S13. MbB and MbB intermediate accumulation in *N. benthamiana* co-expressing CcABCCs. Leaves of *N. benthamiana* transiently expressing genes for respective Mb intermediate production, with CcABCC4A1 (light blue), CcABCC4A2 (dark blue), CcABCC4B (blue hatched), or without (white) transporter co-expression were harvested five days after infiltration. Extracts were analyzed using LC-MS and MbB intermediates were quantified using an external MbA standard curve. Mean and standard error of four biological replicates ($n = 4$) are plotted, and individual data points are shown as dots. The asterisks indicate a statistically significant difference ($^*P < 0.05$) as determined using an unpaired t-test. FW, fresh weight; ns, not significant.

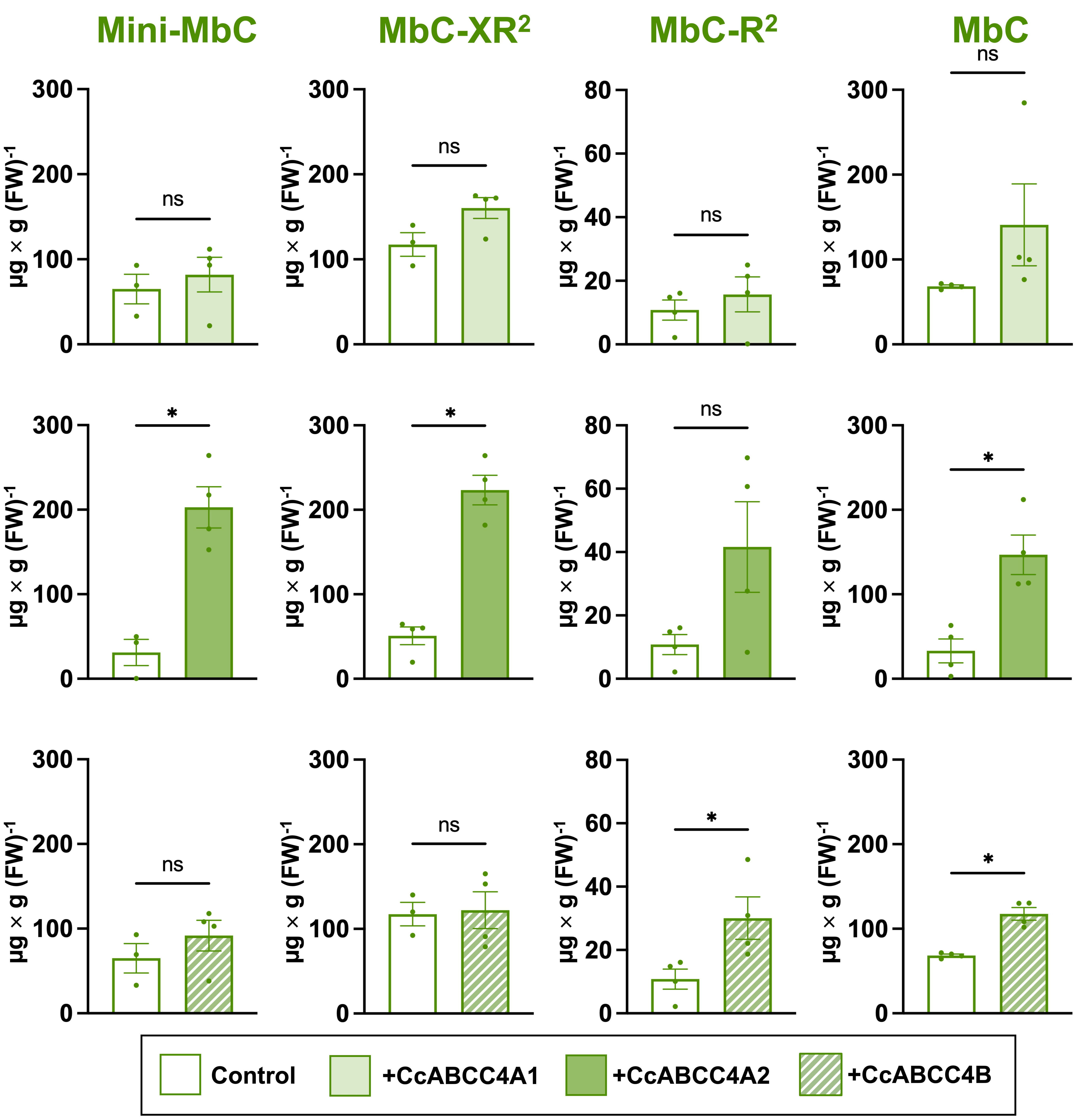

Figure S14. MbC and MbC intermediate accumulation in *N. benthamiana* co-expressing CcABCCs. Leaves of *N. benthamiana* transiently expressing genes for respective Mb intermediate production, with CcABCC4A1 (light green), CcABCC4A2 (dark green), CcABCC4B (green hatched), or without (white) transporter co-expression were harvested five days after infiltration. Extracts were analyzed using LC-MS and total respective MbC intermediates were quantified using an external MbA standard curve. Mean and standard error of four biological replicates (n = 4) are plotted, and individual data points are shown as dots. The asterisks indicate a statistically significant difference (*P <0.05) as determined using an unpaired t-test. FW, fresh weight; ns, not significant.

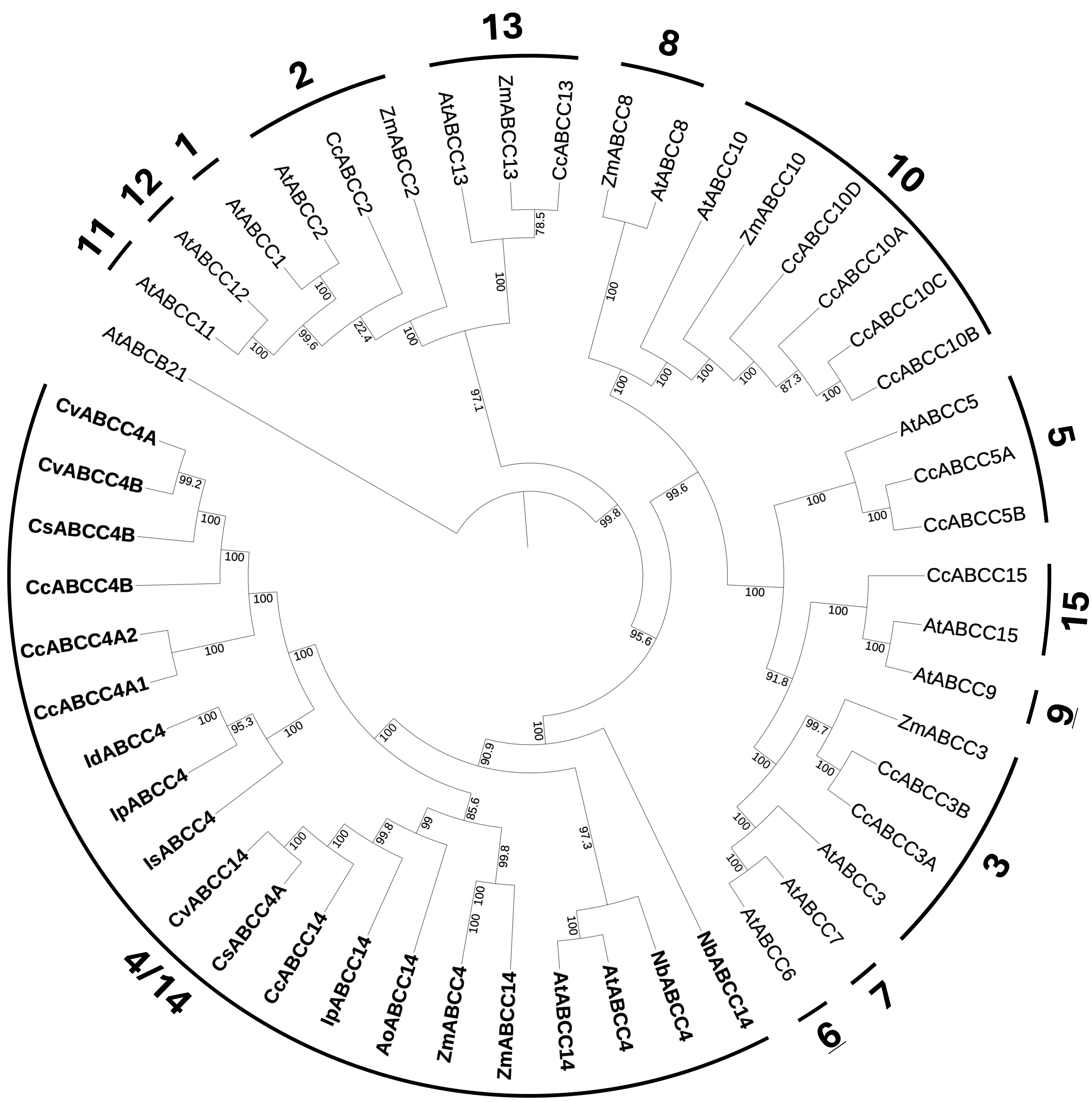

Figure S15. Cladogram of ABCC transporters from montbretia and other plants. Predicted amino acid sequences of montbretia ABCC transporters identified from a corm transcriptome were aligned with ABCC transporters from other plant species using the MUSCLE algorithm in Geneious Prime. The maximum-likelihood phylogeny was constructed using IQTree and visualized using iTOL. All CcABCC transporters were manually annotated and a reciprocal BLASTP against a nonredundant protein database (NCBI) was used to identify and name montbretia homologs based on their closest relatives. Subclades are labeled with their respective numbers on the outside of the cladogram. AtABCB21 was used to root the tree. Bootstrap values (ultrafast) are shown at each node. ABCC transporters characterized in this work are in bold. Amino acid sequences and accession numbers for each sequence can be found in Data S3. *At, Arabidopsis thaliana; Ao, Asparagus officinalis; Cc, Crocosmia x crocosmiiflora; Cs, Crocus sativus; Cv, Crocus vernus; Id, Iris domestica; Ip, Iris pallida; Is, Iris sibirica; Nb, Nicotiana benthamiana; Osi/j, Oryza sativa indica/japonica; Zm, Zea mays.*

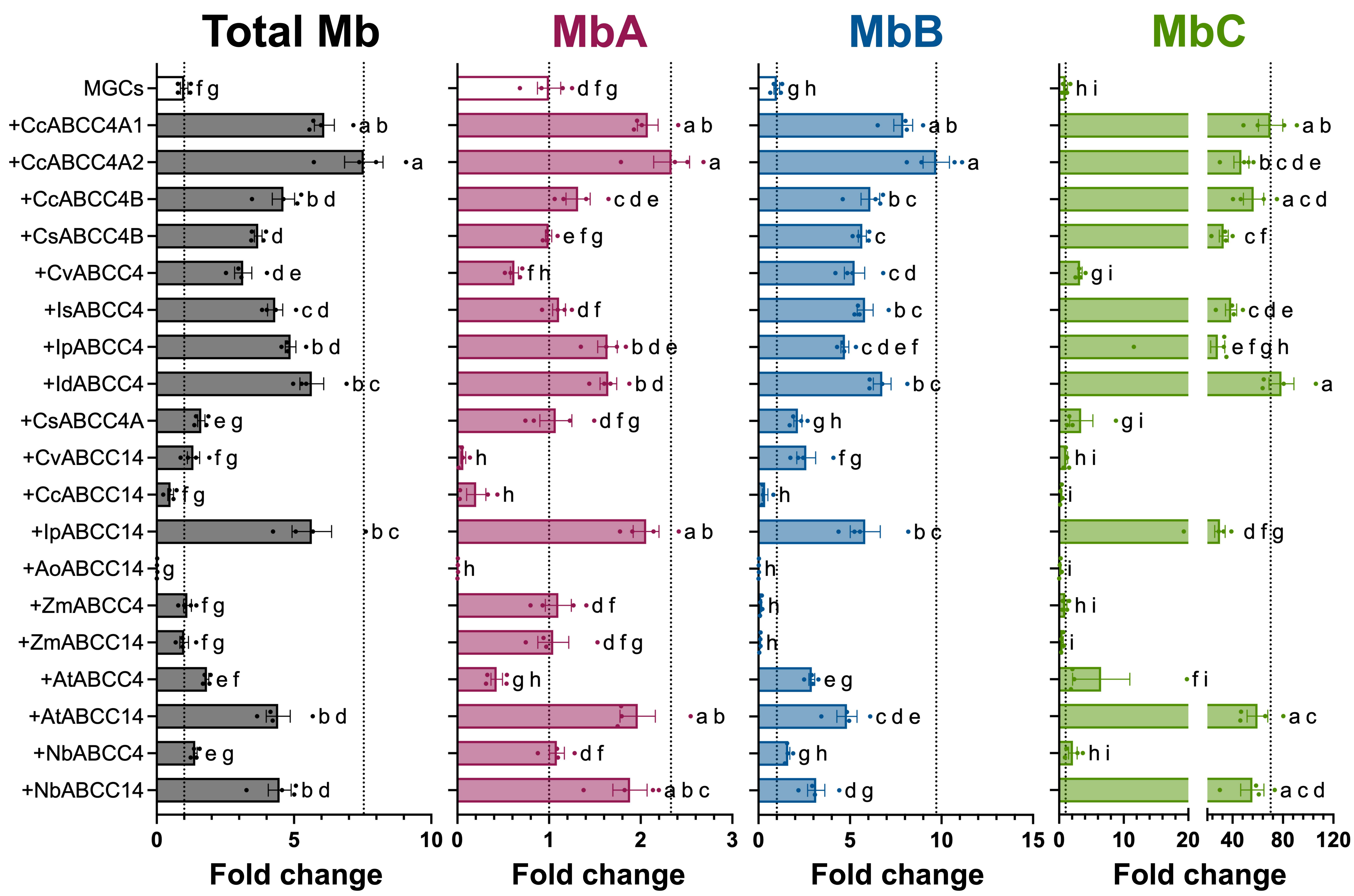

Figure S16. Changes of montbretin levels in *N. benthamiana* upon co-expression of an ABCC transporter with montbretin biosynthetic genes. Leaves of *N. benthamiana* transiently expressing montbretin biosynthetic genes with and without co-expressing a transporter gene were harvested five days after infiltration. Extracts were analyzed using LC-MS and total Mb levels (MbA+MbB+MbC; MbA, MbB, and MbC (from left to right) were quantified using an external MbA standard curve. Mb levels are depicted as fold changes relative to levels in plants not co-expressing a transporter (MGCs), which were set to 1. Data represents the average fold change based on measurements of four biological replicates (n = 4), and individual data points are shown as dots. Different letters indicate statistically significant differences between groups (one-way ANOVA followed by Tukey's HSD post hoc test, $P < 0.05$). Dotted lines indicate the control levels at fold change 1 (left) and the fold change upon co-expression of CcABCC4A2 (right). Mb, montbretin; MGC, multi gene construct; *At, Arabidopsis thaliana; Ao, Asparagus officinalis; Cc, Crocosmia x crocosmiiflora; Cs, Crocus sativus; Cv, C. vernus; Id, Iris domestica; Ip, I. pallida; Is, I. sibirica; Nb, Nicotiana benthamiana; Zm, Zea mays.*

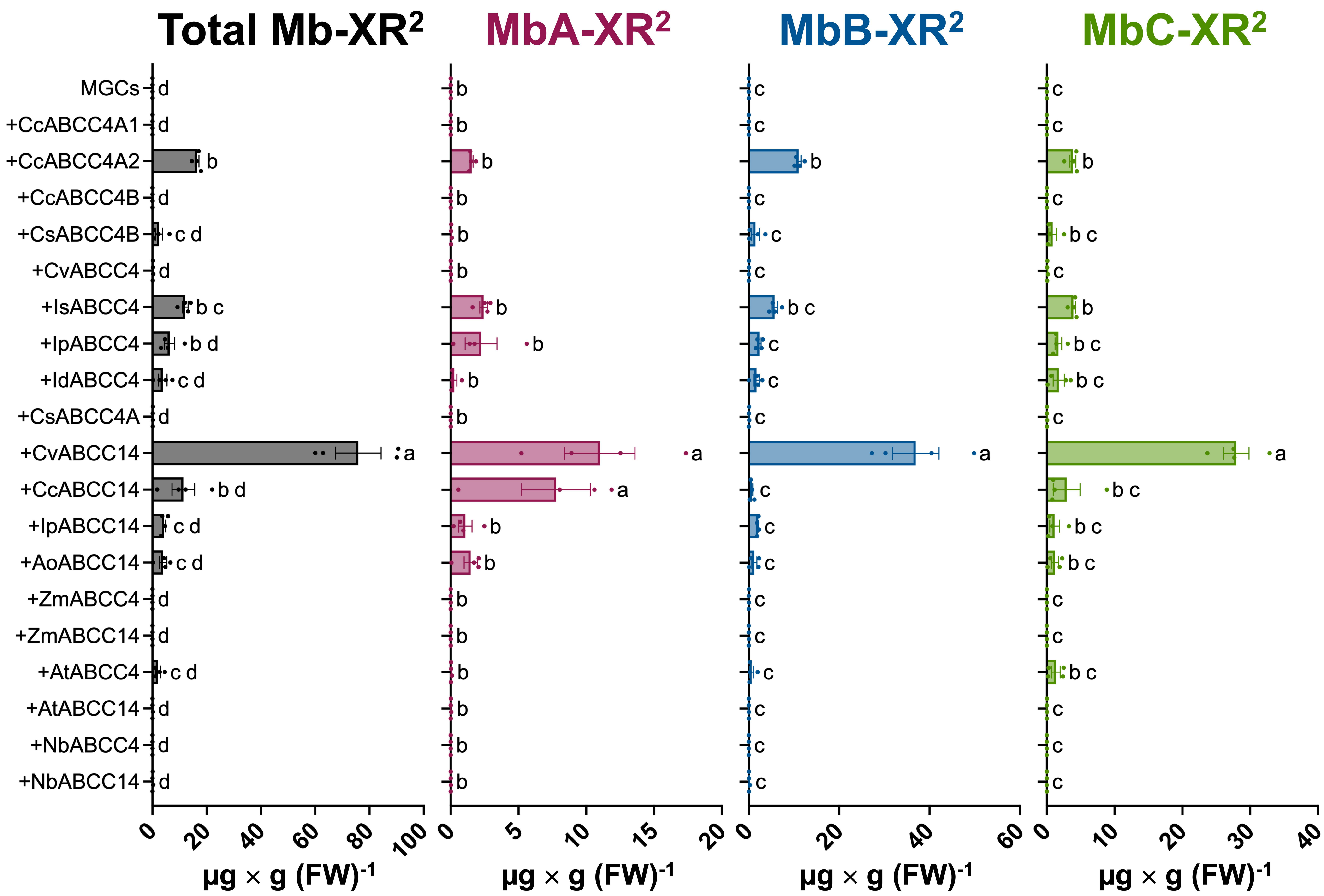

Figure S17. Changes of Mb-XR² levels in *N. benthamiana* upon co-expression of an ABCC transporter. Leaves of *N. benthamiana* transiently expressing Mb biosynthetic genes with and without co-expressing a transporter gene were harvested five days after infiltration. Extracts were analyzed using LC-MS and total Mb-XR² levels (MbA-XR²+MbB-XR²+MbC-XR²) MbA-XR², MbB-XR², and MbC-XR² were quantified using an external MbA standard curve. Data represents the average based on measurements of four biological replicates (n = 4), and individual data points are shown as dots. Different letters indicate statistically significant differences between groups (one-way ANOVA followed by Tukey's HSD post hoc test, $P < 0.05$). Mb, montbretin; *At, Arabidopsis thaliana; Ao, Asparagus officinalis; Cc, Crocosmia x crocosmiiflora; Cs, Crocus sativus; Cv, C. vernus; Id, Iris domestica; Ip, I. pallida; Is, I. sibirica; Nb, Nicotiana benthamiana; Zm, Zea mays.*
