# Supplementary material for: Tonoplast‐Localized ABCC Transporters Enhance Heterologous Production of the Antidiabetic Montbretin A and Its Analogues in Nicotiana benthamiana
Source: Plant Biotechnol J. 2026 Sep 28;24(11):5991–6006. doi: 10.1111/pbi.70765 (PMC13618170; doi:10.1111/pbi.70765)
Supplement: Supplementary file 2 [file PBI-24-5991-s001.zip › pbi70765-sup-0004-FilesS1-S5.docx]

**File S1** Amino acid alignment of CcABC transporters used for construction of the phylogeny shown in Figure S1

**File S2** Amino acid alignment of CcABCC transporters used for construction of the phylogeny shown in Figure 1A

**File S3** Amino acid alignment of CcMATE transporters used for construction of the phylogeny shown in Figure S2

**File S4** Amino acid alignment of ABCC transporters used for construction of the phylogeny shown in Figure S15

**File S5** Amino acid alignment of ABCC4/14 transporters used for construction of the phylogeny shown in Figure 6A
